# Nanobodies targeting norovirus capsid reveal functional epitopes and potential mechanisms of neutralization

**DOI:** 10.1371/journal.ppat.1006636

**Published:** 2017-11-02

**Authors:** Anna D. Koromyslova, Grant S. Hansman

**Affiliations:** 1 Schaller Research Group at the University of Heidelberg and the DKFZ, Heidelberg, Germany; 2 Department of Infectious Diseases, Virology, University of Heidelberg, Heidelberg, Germany; North Carolina State University, UNITED STATES

## Abstract

Norovirus is the leading cause of gastroenteritis worldwide. Despite recent developments in norovirus propagation in cell culture, these viruses are still challenging to grow routinely. Moreover, little is known on how norovirus infects the host cells, except that histo-blood group antigens (HBGAs) are important binding factors for infection and cell entry. Antibodies that bind at the HBGA pocket and block attachment to HBGAs are believed to neutralize the virus. However, additional neutralization epitopes elsewhere on the capsid likely exist and impeding the intrinsic structural dynamics of the capsid could be equally important. In the current study, we investigated a panel of Nanobodies in order to probe functional epitopes that could trigger capsid rearrangement and/ or interfere with HBGA binding interactions. The precise binding sites of six Nanobodies (Nano-4, Nano-14, Nano-26, Nano-27, Nano-32, and Nano-42) were identified using X-ray crystallography. We showed that these Nanobodies bound on the top, side, and bottom of the norovirus protruding domain. The impact of Nanobody binding on norovirus capsid morphology was analyzed using electron microscopy and dynamic light scattering. We discovered that distinct Nanobody epitopes were associated with varied changes in particle structural integrity and assembly. Interestingly, certain Nanobody-induced capsid morphological changes lead to the capsid protein degradation and viral RNA exposure. Moreover, Nanobodies employed multiple inhibition mechanisms to prevent norovirus attachment to HBGAs, which included steric obstruction (Nano-14), allosteric interference (Nano-32), and violation of normal capsid morphology (Nano-26 and Nano-85). Finally, we showed that two Nanobodies (Nano-26 and Nano-85) not only compromised capsid integrity and inhibited VLPs attachment to HBGAs, but also recognized a broad panel of norovirus genotypes with high affinities. Consequently, Nano-26 and Nano-85 have a great potential to function as novel therapeutic agents against human noroviruses.

## Author summary

We determined the binding sites of six novel human norovirus specific Nanobodies (Nano-4, Nano-14, Nano-26, Nano-27, Nano-32, and Nano-42) using X-ray crystallography. The unique Nanobody recognition epitopes were correlated with their potential neutralizing capacities. We showed that one Nanobody (Nano-26) bound numerous genogroup II genotypes and interacted with highly conserved capsid residues. Four Nanobodies (Nano-4, Nano-26, Nano-27, and Nano-42) bound to occluded regions on the intact particles and impaired normal capsid morphology and particle integrity. One Nanobody (Nano-14) bound contiguous to the HBGA pocket and interacted with several residues involved in binding HBGAs. We found that the Nanobodies delivered multiple inhibition mechanisms, which included steric obstruction, allosteric interference, and disruption of the capsid stability. Our data suggested that the HBGA pocket might not be an ideal target for drug development, since the surrounding region is highly variable and inherently suffers from lack of conservation among the genetically diverse genotypes. Instead, we showed that the capsid contained other highly susceptible regions that could be targeted for virus inhibition.

## Introduction

Human norovirus is recognized as the most important cause of outbreaks of acute gastroenteritis [1]. The virus is a non-enveloped single-stranded RNA virus within the *Caliciviridae* family. The human norovirus genome contains three open reading frames (ORFs), where ORF1 encodes non-structural proteins, ORF2 encodes the capsid protein (VP1), and ORF3 encodes the minor capsid protein (VP2). The virion comprises of 90 VP1 dimers that form an icosahedral particle (T = 3) 35–40 nm in diameter [2,3]. The VP1 can be expressed in insect cells and self-assembles into virus-like particles (VLPs) morphologically similar to native virions [4]. Smaller icosahedral particles (15–25 nm, T = 1), presumably composed of 30 VP1 dimers, can also self-assemble in insect cells and were found in patient stool specimens [5,6]. The X-ray crystal structure of norovirus native-size VLPs showed that the VP1 can be divided into shell (S) and protruding (P) domains that are connected via a flexible hinge [3]. The S domain forms the scaffold of the capsid, while the surface exposed P domains contain the main determinants of antigenicity and host binding epitopes.

Noroviruses are genetically diverse and can be divided into seven genogroups (GI-GVII) that are further subdivided into numerous genotypes [7]. The GII genotype 4 (GII.4) includes most epidemic and pandemic strains, while GII.17 was recently attributed with major outbreaks in East Asia [8]. Norovirus illness is typically self-limiting and usually subsides in several days. However, chronic infections in vulnerable individuals, such as the young and elderly, can lead to additional complications and even death [9–11]. Currently there are no available vaccines or antiviral treatments for human noroviruses, despite their discovery over four decades ago [2].

Recently, two cell culture systems have shown that human norovirus can replicate in B-cells or stem cell-derived human enteriods [12,13]. However, norovirus pathogenesis is still poorly understood and the interaction with the host receptor(s) is unclear. Nevertheless, histo-blood group antigens (HBGAs) have been shown to be important binding factors for human norovirus infections [14–17]. HBGAs are found as soluble antigens in saliva and are expressed on epithelial cells, which suggest that noroviruses may encounter HBGAs several times during the course of the infection. Soluble HBGAs may interact with virion particles prior attachment to cells [13] or function as binding factors on cell surfaces [12]. Until recently, the norovirus capsid was presumed to bind two HBGA molecules per VP1 dimer, however two additional HBGA binding sites were identified on the VP1 dimer, indicating that the interaction with HBGAs is rather complex [18,19].

Interestingly, the presence of serum antibodies that block norovirus binding to HBGAs has been associated with a decreased risk of infection and illness [12,20,21]. Moreover, in a recent enteroid norovirus replication system inhibition in the blocking assay was correlated with neutralization in cell culture. A recent study suggested that antibodies targeting the HBGA pocket could inhibit norovirus replication by steric interference with the GI.1 HBGA pocket [22]. A number of other studies have identified norovirus-specific monoclonal antibodies (mAbs) and single chain variable domains (VHH or Nanobodies) that could block norovirus VLP binding to HBGAs [20,23–27]. However, most of these antibodies and Nanobodies are genotype specific, which limits their therapeutic potential [28].

Apart from the HBGA binding site, other neutralizing epitopes likely exist. For example, upon binding to cell receptors, picornaviruses, which are structurally similar to noroviruses, initiate multiple structural rearrangements from the mature capsid to expanded intermediate forms, leading to externalization of the internal polypeptide, membrane fusion and release of viral RNA [29]. Neutralizing Nanobodies that interfere with the conformational rearrangement of the capsid were recently reported for poliovirus [30]. In that study, Nanobodies were used to trap transitional conformations of the viral capsid, which occur during cell entry and are required for the receptor binding.

There is still limited information on norovirus particle attachment to cell surfaces and rearrangements during cell entry. Defining the structural dynamics of the norovirus particles during an infection could show transient conformations related to specific functions in the virus life cycle. These snapshots of the particle dynamics could be obtained from the reconstruction of the capsid protein complexes with antibody fragments or Nanobodies. Moreover, the structural analysis could offer insights into vulnerable regions on the capsid that could be targeted by inhibitors. Indeed, we recently discovered that a human norovirus specific Nanobody (termed Nano-85) bound to intact norovirus VLPs and the Nanobody binding interaction caused the VLPs to disassemble [31]. Our results suggested that the Nano-85 binding epitope might represent a vulnerable region on the capsid that is important for the structural integrity.

In the current study, we analyzed a novel panel of norovirus-specific Nanobodies in order to identify other vulnerable regions. The Nanobody binding epitopes were determined using X-ray crystallography and the specific binding interactions were correlated with a surrogate neutralization assay. We found that Nanobody binding could trigger capsid deformation and increase proteolytic degradation of capsid protein, ultimately exposing viral RNA. Our new findings showed that norovirus particles have vulnerable epitopes that were indispensable for capsid assembly, structural integrity and HBGA attachment.

## Results

We analyzed six new Nanobodies (Nano-4, Nano-14, Nano-26, Nano-27, Nano-32, and Nano-42), which belonged to six groups based on the sequence similarity. The amino acid sequence identity ranged from 65 to 80%, with most sequence variations located in CDR regions. Nano-32 and Nano-4 had exceptionally long CDR3 loops, with 29 and 18 residues, respectively. Nano-32 had an additional disulfide bridge connecting CDR2 and CDR3. Nanobodies with variable CDR loops were expected to bind to distinct epitopes on the capsid.

### Binding specificities

**Fig 1:**
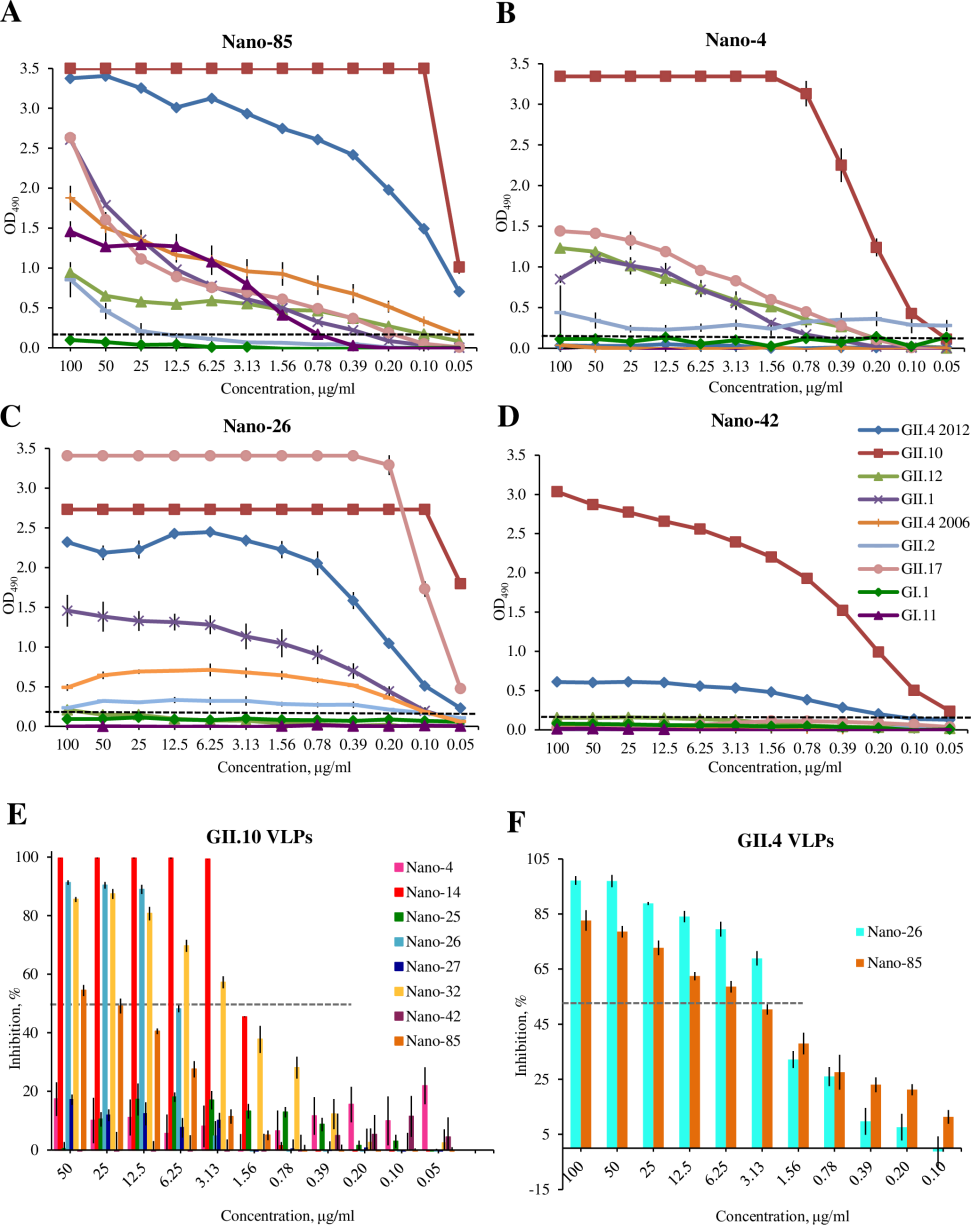
Nanobodies cross-reactivites and inhibition of VLP attachment to PGM. Nanobody cross-reactivities were analyzed using a panel of GII and GI noroviruses in direct ELISA. P domains, 15 μg/ml, (GII.1, GII.2, GII.4, GII.10, GII.12, GII.17) or VLPs, 4 μg/ml, (GI.1 and GI.11) were detected with a panel of serially diluted Nanobodies (A) Nano-85, (B) Nano-4 (C) Nano-26 (D) Nano-42. Nano-85 exhibited the broadest reactivity range and detected all GII noroviruses at 0.4 μg/ml or less and cross-reacted strongly with GI.11 VLPs (<0.8 μg/ml). Nano-26 recognized GII.1, GII.2, GII.4, GII.10, GII.12, and GII.17 P domains. Nano-4 bound GII.10, GII.17, GII.12, and GII.1 P domains. Nano-42 could only detect GII.10 and GII.4 2012, whereas Nano-27, Nano-32, Nano-14 did not cross-react with any examined P domains. All experiments were performed in triplicate (error bars are shown) and the cutoff was set at an OD_490_ of 0.15 (dashed line). (E, F) PGM blocking assay was used as a surrogate neutralization assay. GII.10 VLPs were pretreated with serially diluted Nanobodies and added on PGM coated plates. (E) Inhibition of GII.10 VLPs binding to PGM. Nano-14 and Nano-32 inhibited 50% (IC_50_) of the binding at 1.7 to 2.6 μg/ml, respectively. For Nano-26, the IC_50_ value was 6.6 μg/ml. Nano-85 showed only weak blocking potential. (F) Inhibition of GII.4 VLPs binding to PGM. Nano-85 and Nano-26 blocked the binding with IC_50_ values of 2.2 μg/ml and 2.5 μg/ml, respectively. Binding is expressed as a percentage of the untreated VLP binding (100%). 50% inhibition is shown as a dashed line. All experiments were performed in triplicate (error bars are shown).

The Nanobody binding specificities were initially confirmed with the immunization antigen (i.e., GII.10 VLPs) and the corresponding GII.10 P domain (S1 Fig). All six Nanobodies bound to the GII.10 VLPs and P domain, which indicated that the S domain did not contain any Nanobody binding epitopes. Nano-42, Nano-14, Nano-26, and Nano-4 showed the strongest binding capacities (0.02–0.2 μg/ml), whereas Nano-27 and Nano-32 had lower binding ability (1.5 and 0.2 μg/ml, respectively). Following these results, the cross-reactivities were analyzed with a panel of VLPs and P domains from various GI (GI.1 and GI.11) and GII (GII.1, GII.2, GII.4 2006 and 2012, GII.10, GII.12, and GII.17) genotypes (Fig 1). Nano-85 exhibited the broadest recognition range, detecting GI.11 VLPs and numerous GII P domains. Nano-26 also showed broad reactivity, detecting all GII genotypes. Nano-4 and Nano-42 showed limited cross-reactivity, while Nano-27, Nano-32, and Nano-14 were GII.10 specific (S1 Fig).

### HBGA blocking properties of Nanobodies

In order to determine the HBGA blocking potential of the Nanobodies, a surrogate neutralization assays were performed using GII.10 and GII.4 VLPs. Three Nanobodies (Nano-14, Nano-32, and Nano-26) inhibited the binding of GII.10 VLPs to PGM in a dose-dependent manner (IC_50_ = 1.7 to 6.6 μg/ml) (Fig 1E). Similarly, Nano-14, Nano-26, and Nano-32 inhibited binding to A-type saliva (IC_50_ = 0.3 to 3.1 μg/ml) and B-type saliva (IC_50_ = 1.1 to 4.3 μg/ml) (S2A and S2B Fig). Nano-85 was relatively ineffective in blocking the GII.10 VLPs to PGM or B-type saliva (IC_50_ > 70 μg/ml) and weakly blocked GII.10 VLPs to A-type saliva (IC_50_ = 12 μg/ml). Nano-4, Nano-25, Nano-27, and Nano-42 demonstrated no inhibition of GII.10 VLPs. Additionally, both Nano-26 and Nano-85 blocked GII.4 VLPs from binding to PGM (Fig 1F) (IC_50_ 2.4 μg/ml and 3.1 μg/ml, respectively) and B-type saliva (IC_50_ 0.7 μg/ml and 1.2 μg/ml, respectively) (S2C Fig). To demonstrate that Nano-26 and Nano-85 specifically inhibit VLP binding to HBGAs present in PGM and saliva, a blocking assay using synthetic HBGAs was performed (S2D Fig). Nano-26 and Nano-85 blocked GII.4 VLPs from binding to synthetic B-tri saccharide with IC_50_ ranging between 1 μg/ml to 10 μg/ml. Nano-4 and Nano-42 did not inhibit GII.4 VLPs from binding to PGM.

### Thermodynamic properties

The thermodynamic properties of Nanobodies binding to GII.10 P domains were analyzed using ITC (Table 1). Most of the Nanobodies (Nano-4, Nano-14, Nano-26, Nano-27, and Nano-42) exhibited exothermic binding with nanomolar affinities (S3 Fig). The binding reaction was driven by a large enthalpy change and was characterized with unfavorable entropy of the binding. This suggested that the net formation of non-covalent bonds between the Nanobody and the P domain was a major contributor to the binding. The stoichiometry indicated the binding of one Nanobody molecule per P domain monomer for all Nanobodies, except Nano-14, where the ratio of P domain:Nanobody was 2:1. Nano-32 binding was characterized by a positive enthalpy change associated with endothermic type of reaction (S3 Fig). Instead, a large positive entropy was the main contributing factor to the ΔG. These different thermodynamic parameters were likely associated with the distinct binding epitope of Nano-32, as presented below.

**Table 1:** Thermodynamic properties of Nanobody binding to the P domain.

	ΔK_d_	ΔH	ΔS	ΔG
**GII.10-Nano-4**	3.50E-08 (2.72E-08)	-1.20E+04 (180)	-3.2 (2.7)	-1.10E+04 (592)
**GII.10-Nano-14**	1.04E-08 (2.60E-09)	-2.20E+04 (190)	-36.1 (7.5)	-1.10E+04 (110)
**GII.10-Nano-26**	3.20E-09 (1.22E-09)	-1.00E+04 (95)	4.8 (1.4)	-1.20E+04 (270)
**GII.10-Nano-27**	9.50E-09 (1.90E-10)	-8.10E+03 (334)	9.5 (1.1)	-1.10E+04 (19)
**GII.10-Nano-32**	6.00E-08 (2.10E-08)	5.00E+03 (132)	49.8 (0.7)	-9.90E+03 (245)
**GII.10-Nano-42**	8.09E-10 (7.36E-10)	-9.84E+03 (80)	9.1 (1.9)	-1.30E+04 (500)

Titrations were performed at 25°C by injecting consecutive aliquots of 100 μM of Nanobodies into 15 μM of P domain. The binding isotherm was calculated using a single binding site model. The binding constants, K_d_ (dissociation constant, M), ΔH (heat change, cal/mole), ΔS (entropy change, cal/mole/deg), ΔG (change in free energy, cal/mol). All experiments were performed in triplicate. Standard deviations are shown in brackets.

### X-ray crystal structures of norovirus P domain Nanobody complexes

**Fig 2:**
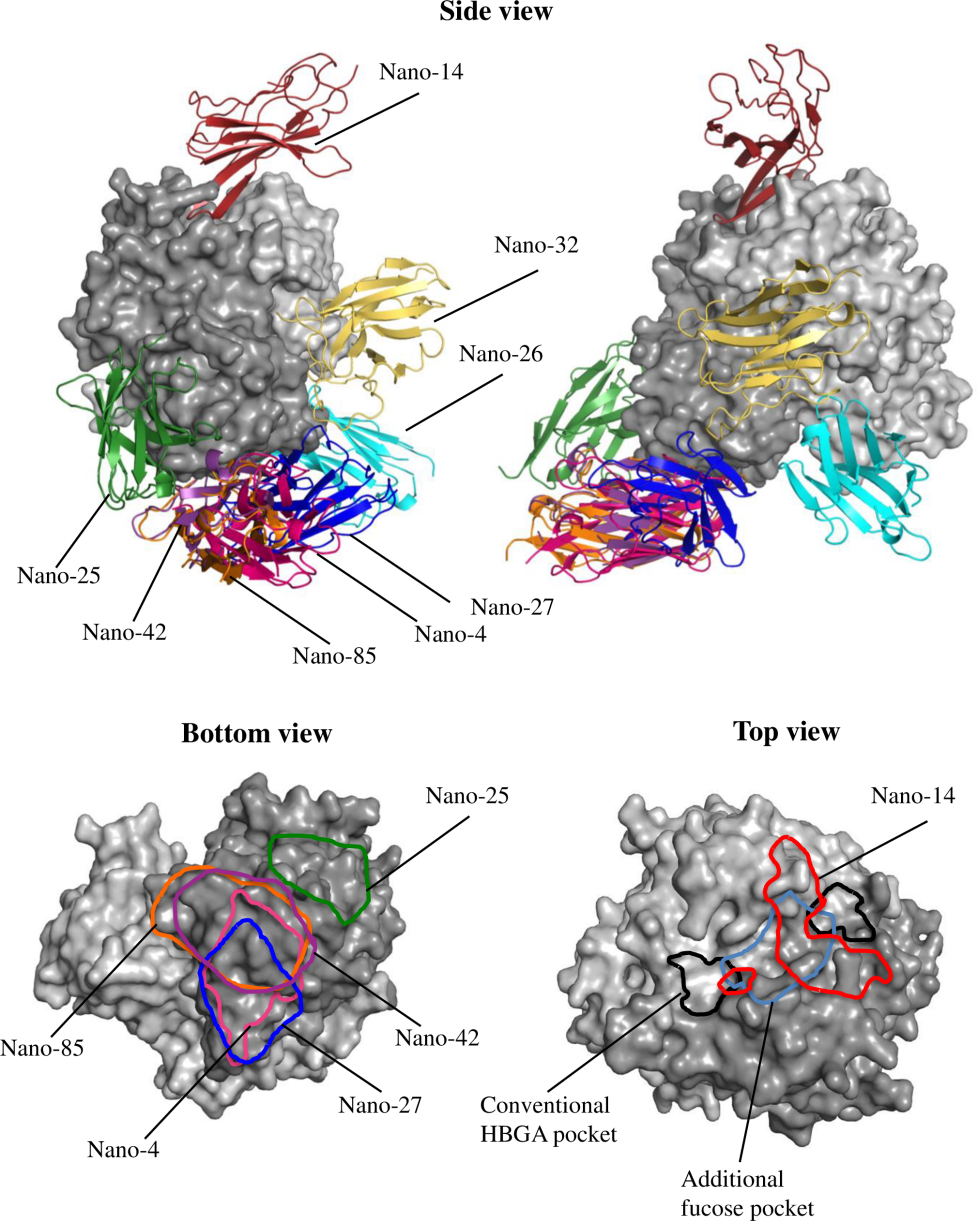
Variety of Nanobody binding sites on the GII.10 domain. The X-ray crystal structures of the P domain-Nanobody complexes were superimposed onto each other. Nano-85 and Nano-25 complex structures were previously published in [31]. GII.10 P domain is colored light gray (chain A) and dark gray (chain B), Nano-14 (red), Nano-42 (dark purple), Nano-32 (yellow), Nano-27 (blue), Nano-26 (cyan), Nano-4 (pink), Nano-85 (orange), and Nano-25 (dark green). HBGA binding sites are marked black and newly identified additional fucose binding sites are marked blue. One Nanobody bound on the top of the P domain (Nano-14), two Nanobodies bound on the side (Nano-32 and Nano-25) and five Nanobodies bound on the bottom (Nano-85, Nano-4, Nano-26, Nano-42 and Nano-27). Nano-32, Nano-26 and Nano-14 were involved in a dimeric interaction with the P domain, whereas the binding of Nano-4, Nano-25, Nano-27, and Nano-85 was monomeric. Nanobody binding footprints are marked on the bottom and top of the P domain.

The structures of GII.10 P domain in complex with Nano-14, Nano-26, Nano-27, Nano-32, and Nano-42 were solved using X-ray crystallography (Table 2). Additionally, the X-ray crystal structure of GII.17 P domain with Nano-4 was determined in order to explain Nanobody cross-reactivity binding interactions at the atomic level. We also solved a double complex structure of GII.10 P domain with Nano-26/Nano-85, which permitted a higher resolution than the GII.10 P domain and Nano-26 complex alone, and explained how two distinct Nanobodies bound simultaneously to one P dimer. The overall structure of the P domains in all complex structures was reminiscent of unbound P domain with limited structural changes observed upon binding of the Nanobodies. All Nanobodies had typical immunoglobulin fold and interacted with the P domain primarily with CDR loops. The electron densities for Nano-4, Nano-32, Nano-26, Nano-27, and Nano-42 were well resolved, whereas for Nano-14, the distant part of a Nanobody close to the two-fold crystallographic symmetry axis was partially disordered. Overall, we could separate six Nanobodies into three distinct binding regions on the P domain: termed top, side, and bottom. Nano-4, Nano-26, Nano-27, and Nano-42 bound to the bottom; Nano-32 bound to the side; and Nano-14 bound on the top of the P domain (Fig 2 and Table 3). The binding sites of Nano-4 and Nano-27 partially overlapped the Nano-85 binding site. Moreover, Nano-42 bound with almost identical orientation as Nano-85.

**Table 2:** Data collection and refinement statistics for P domain Nanobody complex structures.

	Nano-4 GII.17 P domain	Nano-14 GII.10 P domain	Nano-26 Nano-85 GII.10 P domain	Nano-27 GII.10 P domain	Nano-32 GII.10 P domain	Nano-42 GII.10 P domain
PDB	5O02	5OMM	5O04	5OMN	5O03	5O05
Data collection						
Space group	C121	C121	C121	P6222	P4_1_2_1_2	P2_1_2_1_2_1_
Cell dimensions						
*a*, *b*, *c* (Å)	118.5 64.0 70.0	97.5 94.2 101.3	167.2 91.5 118.1	167.4 167.4 143.5	109.7 109.7 268.3	87.9 105.4 107.7
α, β, γ (**°**)	90 97.26 90	90 112.70 90	90 127.12 90	90 90 120	90 90 90	90 90 90
Resolution range (Å)	45.1–1.69 (1.80–1.69)*	48.50–1.65 (1.75–1.65)*	47.98–2.30 (2.44–2.30)*	48.34–2.68 (2.78–2.68)*	49.04–2.19 (2.33–2.19)*	47.33–2.00 (2.12–2.00)*
*R*_merge_	6.8 (42.7)*	6.4 (56.1)*	7.9 (59.2)*	15.0 (195.8)*	5.9 (77.3)*	9.02 (79.2)*
*I*/σ*I*	11.5 (2.3)*	16.5 (2.8)*	10.7 (2.3)*	20.6 (1.65)*	23.8 (2.7)*	13.1 (2.0)*
Completeness (%)	96.5 (90.2)*	98.0 (95.3)*	91.7 (90.1)*	99.5 (97.0)*	99.8 (98.9)*	99.6 (98.8)*
Redundancy	3.2 (2.8)*	4.9 (4.8)*	2.8 (2.7)*	19.3 (18.7)*	10.6 (7.6)*	4.5 (4.6)*
CC1/2	99.7 (84.9)	99.9 (80.6) *	99.6 (76.3)	99.9 (74.4)	99.9 (92.5)	99.8 (74.9)
Refinement						
Resolution range (Å)	45.06–1.77	48.50–1.7	47.98–2.30	48.34–2.68	49.04–2.19	48.76–2.63
No. of reflections	55890	99684	58206	33588	84286	68145
*R*_work_/*R*_free_	15.1/18.2	16.5/19.3	21.2/24.1	23.5/26.2	17.2/19.3	18.0/22.1
No. of atoms	4034	5957	8217	3218	7011	6995
Protein	3490	5440	8064	3218	6706	6425
Ligand/ion	29	100	72	0	70	156
Water	515	417	81	0	887	414
Average *B* factors (Å^2^)						
Protein	19.10	26.30	52.30	68.20	63.40	40.40
Ligand/ion	43.60	36.20	56.80	0	75.30	53.10
Water	33.00	29.30	38.20	0	54.40	40.70
RMSD						
Bond lengths (Å)	0.006	0.008	0.002	0.002	0.005	0.009
Bond angles (**°**)	1.05	1.16	0.67	0.48	0.87	1.08

Each data set was collected from single crystals, respectively.

*Values in parentheses are for the highest-resolution shell.

**Table 3:** Summary of Nanobodies analyzed in this study.

Nanobody	Affinity to GII.10, K_d_	Cross reactivity	PGM blocking	Binding site on the P domain		EM
**Nano-4**	3.50E-08	GII.1, GII.12, GII.17	No	Bottom	Monomeric	Small
**Nano-14**	1.04E-08	No	Yes	Top	Dimeric	Native
**Nano-26**	3.20E-09	GII.1, GII.2, GII.4, GII.17	Yes	Side, bottom	Dimeric	Broken/Small
**Nano-27**	9.50E-09	No	No	Side, low	Monomeric	Small
**Nano-32**	6.00E-08	No	Yes	Side, middle	Dimeric	Aggregated native
**Nano-42**	8.09E-10	GII.4	No	Bottom	Monomeric	Small/Broken
**Nano-85**	3.47E-09	GII.1, GII.2, GII.4, GII.12, GII.17, GI.11	Moderate	Bottom	Monomeric	Broken/Small

To support our structural data and exclude the possibility of less probable orientations derived from the crystal packing we performed competitive ITC measurements. Nano-85 showed no binding to the P domain pre-incubated with Nano-4, Nano-27 and Nano-42, indicating that these Nanobodies competed for the same binding region on the P domain (S4 Fig). On the contrary, when Nano-85 titrations were performed to the P domain pre-mixed with Nano-14 or Nano-26 the binding isotherm was reminiscent of the Nano-85 P domain measurement. These data implied that Nano-14 and Nano-26 bound to sites distinct from Nano-85, whereas other Nanobodies competed with the Nano-85 epitope. Therefore, these P domain Nanobody complex structures clearly represented the precise Nanobody binding epitopes.

### Structure of GII.10 P domain Nano-14 complex

**Fig 3:**
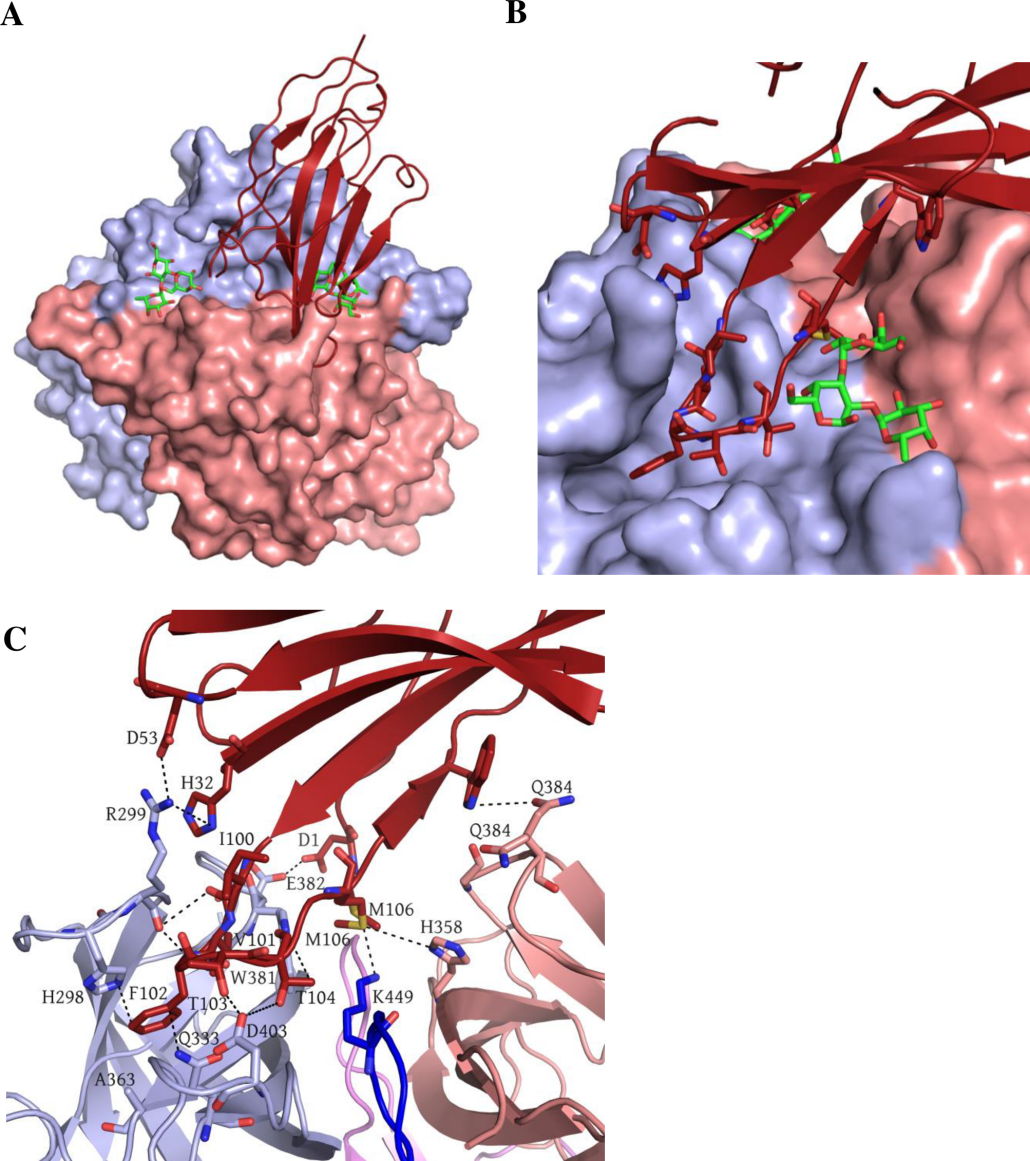
Nano-14 in complex with the GII.10 domain. (A) The X-ray crystal structure of the GII.10 domain Nano-14 complex was determined to 1.7Å resolution. The molecular replacement in C121 space group indicated one P domain dimer and one Nanobody per asymmetric unit. P domain chain A (light blue), chain B (salmon), Nano-14 (red). The Nano-14 bound to the top of the P1 subdomain in the canyon between two monomers. (B) The Nano-14 binding site overlapped with the binding pocket of HBGAs (as an example B-trisaccharide is shown in green sticks) (C) A close-up view of GII.10 P domain and Nano-14 interacting residues. The P domain hydrogen bond interactions included side-chain and main chain interactions from both monomers. R299, W381, K449, D403, and E333 from chain A and Glu384 from chain B formed direct hydrogen bonds with Nano-14: D53, D1, F102, T103, T104, M106, and W109. P domain E382 and R299 were involved in electrostatic interactions with Nano-14 residues D1 and H32. Hydrophobic interactions involved P domain chain A: W381, H298, R299, V361, A363 and Nano-14: F102, I100, V101, M106, and H32. Two additional interactions were observed with P domain chain B residues: direct hydrogen bond with Q384 and Pi-sulfur interaction with H358.

The structure of GII.10 P domain Nano-14 complex was solved to 1.8Å resolution. Nano-14 bound on the top of the P domain in the grove located between the two P domain monomers (Fig 3A and 3B). A vast network of hydrogen bonds was formed between Nano-14 and both P domain monomers. The majority of interactions were built between one P domain monomer (chain A) and CDR3 of the Nano-14 (Fig 3C). Six P domain residues (chain A: Arg299, Trp381, Lys449, Asp403, and Glu333; chain B: Gln384) formed eleven direct hydrogen bonds with Nano-14. Four electrostatic interactions were observed between Nano-14 and the P domain residues Arg299 and Glu382 (chain A). The numerous hydrogen bonds and electrostatic interactions corresponded well with the large negative binding enthalpy (S3 Fig). Five P domain residues (His298, Val361, Ala363, Arg299, and Trp381) were involved in eight hydrophobic interactions with Nano-14. Two additional interactions were observed: P domain His358 (chain B) formed a π-sulfur interaction with Nanobody Met106, whereas P domain Glu333 (chain A) participated in a π donor hydrogen bond with Phe102.

**Fig 4:**
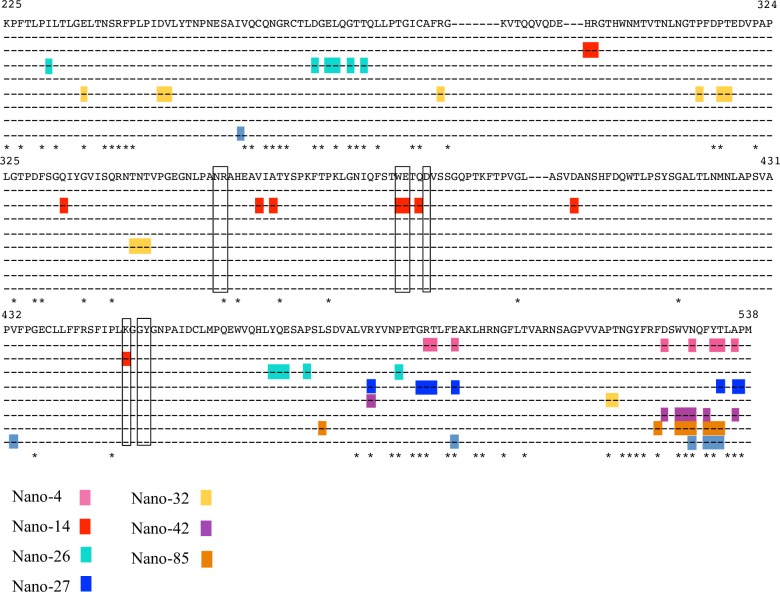
Nanobody binding epitopes on the GII P domain sequence alignment. Eleven different GII genotype P domain sequences were aligned using ClustalX. The GII.10 capsid sequence was used as the consensus sequence, other sequences include GII.1 (U07611), GII.2 (HCU75682), GII.3 (DQ093066), Saga-2006 GII.4 (AB447457), NSW-2012 GII.4 (JX459908), GII.5 (BD011877), GII.6 (BD093064), GII.7 (BD011881), GII.8 (AB039780) GII.10, and GII.12 (AB044366). For clarity only GII.10 residues are shown. The binding epitope of a broadly reactive monoclonal antibody 5B18 MAb (light blue) is shown for the reference. The GII.10 residues interacting with Nano-4 (pink), Nano-14 (red), Nano-25 (dark green), Nano-27 (blue), Nano-32 (yellow), Nano-42 (deep violet), and Nano-85 (orange) are colored accordingly. The asterisks mark conserved amino acids. P domain residues interacting with HBGAs are boxed.

The nine P domain residues involved in Nano-14 binding were predominantly variable (see Fig 4). This finding corresponded nicely with the ELISA data that showed Nano-14 was GII.10 specific. Remarkably, the Nano-14 binding site, which is largely formed by CDR3 loop, extended between two HBGA binding pockets (Fig 2). Such strategic positioning of Nano-14 resulted in steric interference with the two conventional HBGA binding sites and the two newly identified HBGA binding pockets [18]. Moreover, three P domain residues (Trp381, Glu382 and Lys449) were directly involved in binding HBGAs [32] and Nano-14, indicating a direct competition for the HBGA pocket. Importantly, analysis of the Nano-14 binding site with the ELISA blocking data provided a novel structural basis of GII HBGA binding interference (see Fig 1).

### Structure of GII.10 P domain Nano-32 complex

**Fig 5:**
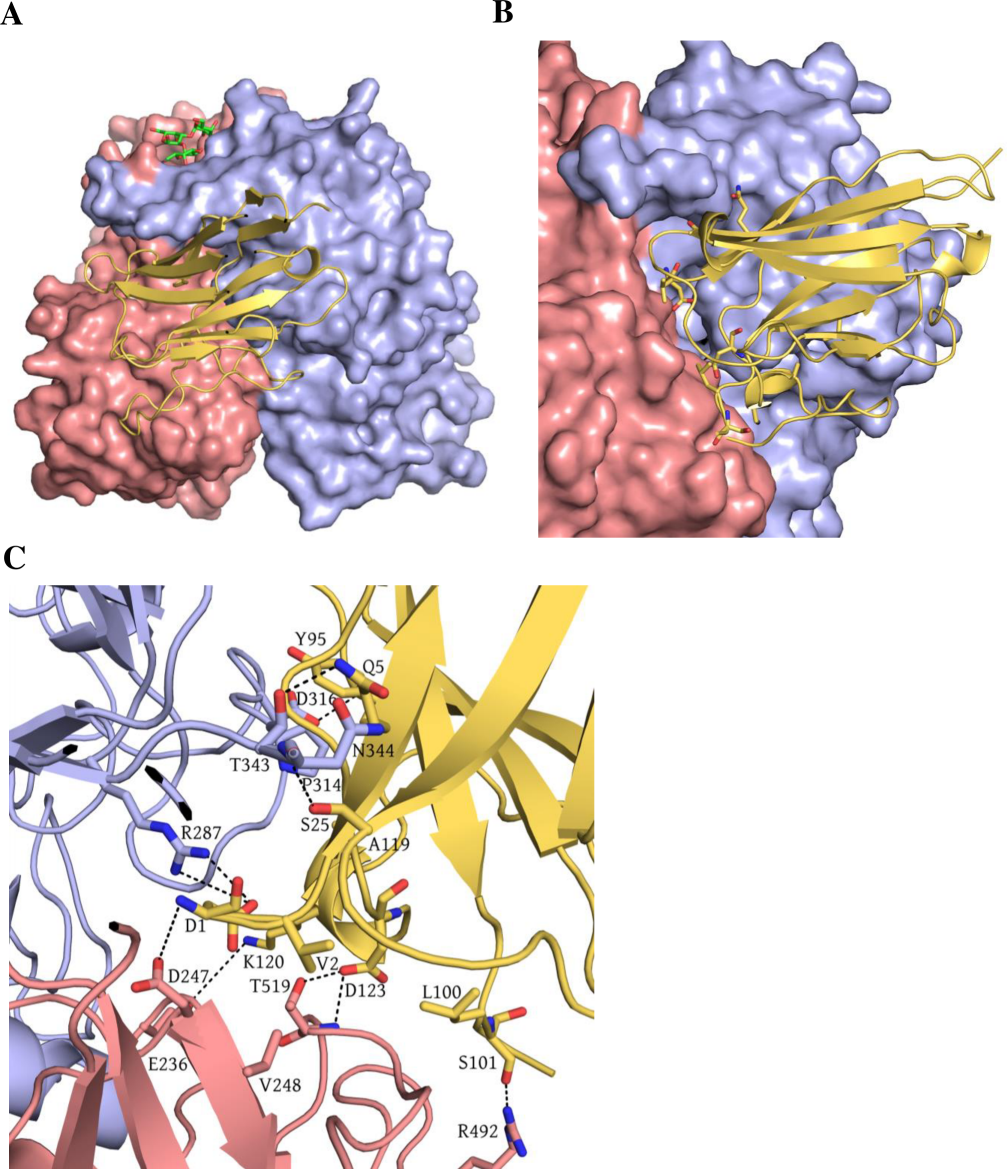
Nano-32 in complex with the GII.10 domain. The X-ray crystal structure of the GII.10 domain Nano-32 complex was determined to 2.1Å resolution. The asymmetric unit cell contained one P domain dimer and two Nanobodies in space group P4_1_2_1_2. The interface with the surface area of 650 Å^2^ was considered biologically relevant. (A) The complex was colored according to Fig 3 with Nano-32 (yellow). (B) The Nano-32 bound to the side of the P1 subdomain in the cleft between two monomers. (C) A close-up view of GII.10 P domain and Nano-32 interacting residues. The P domain hydrogen bond interactions included side chain and main chain interactions from both monomers. Direct hydrogen bonds were formed with chain A: R287, N344, W343, D316 and chain B: R492 and W519 with Nano-32: D1, N5, S25, L45, S101, and D123. Electrostatic interactions formed between P domain chain A: R287, E236, chain B: D247 and Nano-32: D1 and K120. Hydrophobic interactions involved chain A: P314 and chain B: P518, V248 and Nano-32: V2, L45, Y95, and A119.

The Nano-32 binding site was located on the side of the GII.10 P domain in a cleft between two P domain monomers (Fig 5A and 5B). In the P domain Nano-32 complex, several P domain loops were slightly shifted compared to the unliganded P domain (S5 Fig) (chain A: residues 487–491 and 517–522; chain B: residues 309–314, 287–300, and 418–421). Moreover, a P domain loop (residues 343–352) was shifted ~4.3Å from the loop in the unliganded structure. Several residues within this loop were also disordered, suggesting a certain degree of P domain flexibility. The loop containing residues 295–300 was positioned identically in both monomers in contrast to the usual asymmetric orientation in unliganded structure [18]. These conformational rearrangements likely correlated with the major entropy change observed in ITC measurements (Table 1). Nano-32 was essentially held equally with two P domain monomers (Fig 5C). Four P domain residues from chain A (Arg287, Asn344, Trp343, and Asp316) and two residues from chain B (Arg492 and Thr519) formed seven direct hydrogen bounds with Nano-32. Several P domain residues were also involved in electrostatic interactions (chain A: Arg287 and Asp247; chain B: Glu236) and hydrophobic interactions (chain A: Pro314; chain B: Val248 and Pro518). Six P domain residues involved in Nano-32 binding were highly variable and five residues were conserved in GII.4 and GII.10 noroviruses (Fig 4). Although Nano-32 strongly inhibited binding of GII.10 VLPs to HBGAs, none of the residues were shared between the HBGA pockets and the Nano-32 binding site. This result suggested that Nano-32 indirectly interfered with the HBGA pockets or utilized another mechanism to inhibit HBGA binding.

### Structure of GII.17 P domain Nano-4 complex

**Fig 6:**
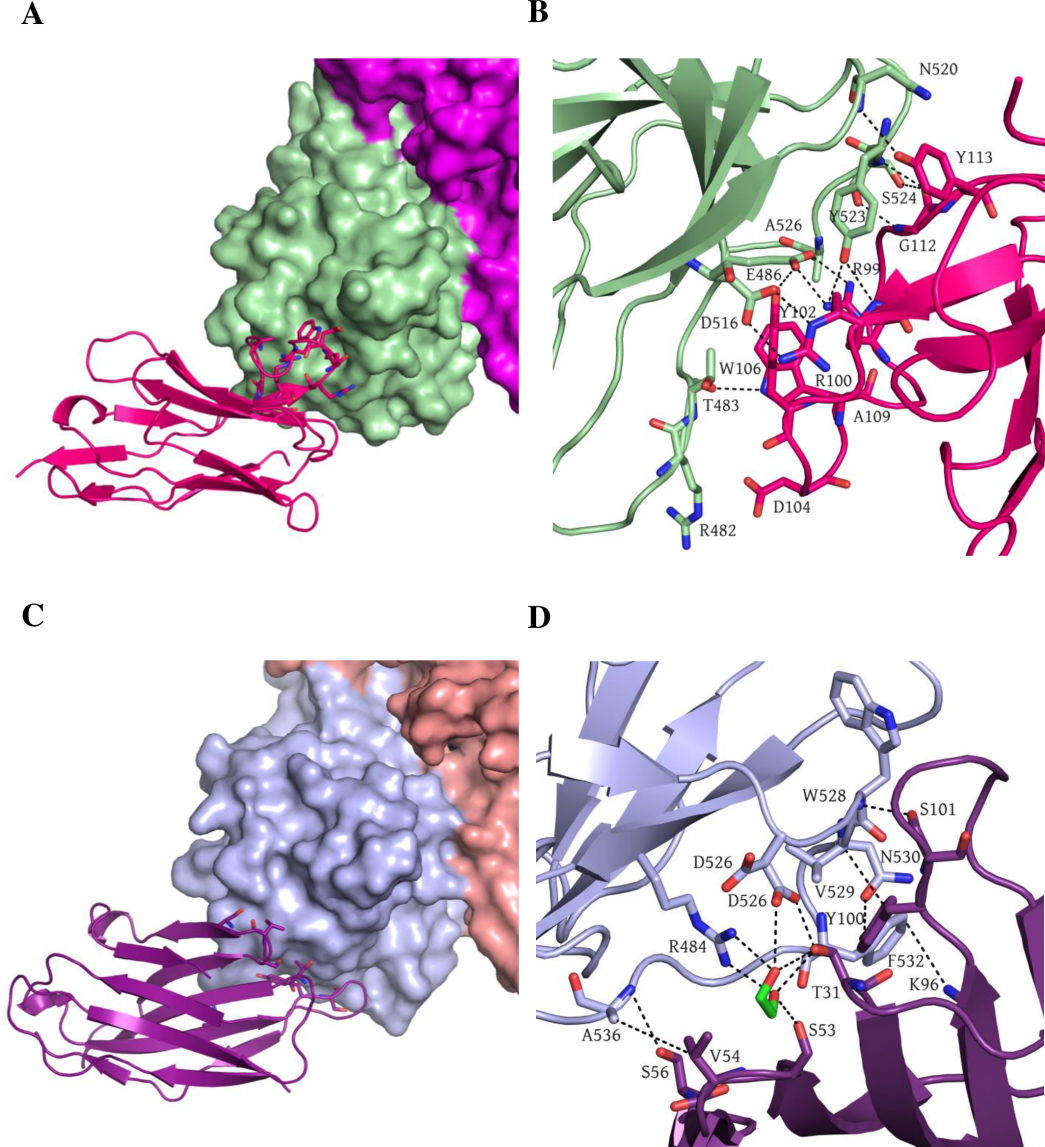
Nano-4 GII.17 domain and Nano-42 GII.10 P domain complex structures. (A, B) The X-ray crystal structure of the GII.17 domain Nano-4 complex was determined to 1.7Å resolution. Unit cell contained one P domain and one Nanobody. Only one relevant interface with a surface area of 532 Å^2^ was found. GII.17 P domain is colored violet (Chain A) and light green (Chain B), Nano-4 is shown in hot pink. A close up view shows the formation of the extended network of hydrogen bonds between P domain residues: T483, E486, D516, N520, Y523, S524 and Nano-4: R99, R100, Y102, T106, G112, and Y113. Two hydrophobic interactions were formed between P domain: Y523, A526 and Nano-4: Y113, A109. Five electrostatic interactions (P domain: R482, E486, D516) contributed to binding to Nano-4: D104, R100. (C, D) The structure of GII.10 P domain and Nano-42 was solved to 2.0Å resolution with the unit cell containing one P domain dimer and two Nanobodies. The Nano-42 binding site had an interface surface area of 621 Å^2^. GII.10 P domain is colored as in Fig 3 and Nano-42 is colored deep purple. Five direct hydrogen bonds involved P domain residues: D526, W528, N530, T534 and Nano-42 residues: T31, Y100, S101, and S56, one electostatic interaction formed between P domain F532 and Nano-42 K96. Two hydrophobic interactions were formed between P domain residues V529, A536 and Nano-42 residues Y100, V54. Ethylene glycol molecule is shown in green sticks and participates in six direct hydrogen bonds with P domain and Nanobody.

We solved the structure of GII.17 P domain Nano-4 complex, since the GII.17 norovirus was of recent clinical concern; and we wanted to analyze the cross-reactive epitopes at the atomic level. According to the ELISA data, Nano-4 bound strongly to the GII.17 VLPs. X-ray data for GII.17 P domain Nano-4 complex was processed to 1.7Å resolution in C121 space group. Nano-4 bound to the bottom of the P domain in close proximity to the previously identified Nano-85 binding site (Fig 2 and Fig 6A and 6B) [31]. An extensive network of direct hydrogen bonds was formed between P domain residues (Thr483, Glu486, Asp516, Asn520, Tyr523, and Ser524) and Nano-4 (Fig 6B). Two P domain residues were involved in hydrophobic interactions (Tyr523 and Ala526) and five electrostatic interactions (Arg482, Glu486, and Asp516) contributed to Nano-4 binding. Only three of nine P domain residues interacting with Nano-4 were variable (Fig 4). The six conserved residues provided a possible explanation for the broad cross-reactivity exhibited with Nano-4 (Fig 1). The Nano-4 binding epitope was located on the opposite side of the HBGA pocket, an observation that is supported by the lack of blocking potential in the surrogate neutralization assay.

### Structure of GII.10 P domain Nano-42 complex

Nano-42 bound on the bottom of the P domain and closely overlapped with Nano-85 binding site (Fig 6C). Five direct hydrogen bonds involved P domain residues (Asp526, Trp528, Asn530, and Thr534) and Nano-42 residues (Fig 6D). Two hydrophobic interactions were formed between P domain residues Val529 and Ala536 and Nano-42 residues Tyr100 and Val54, respectively. Interestingly, the Nanobody was also held by three additional hydrogen bonds mediated by an ethylene glycol molecule. Ethylene glycol interacted with P domain residues Arg484 and Asp526 on one side and Nano-42 residues Thr31 and Ser53 on the other side. Moreover, six water mediated bonds provided additional stabilization of the bound Nano-42. Although Nano-42 binding residues were mainly conserved in GII noroviruses and were identical between GII.4 2006 and 2012 strains, Nano-42 apparently distinguished these two strains in the ELISA cross-reactivity study (Fig 1). In addition, although the binding epitope of Nano-42 was rather similar to that of Nano-85, Nano-42 did not inhibit VLP binding to HBGAs.

### Structure of GII.10 P domain Nano-26 Nano-85 double complex

**Fig 7:**
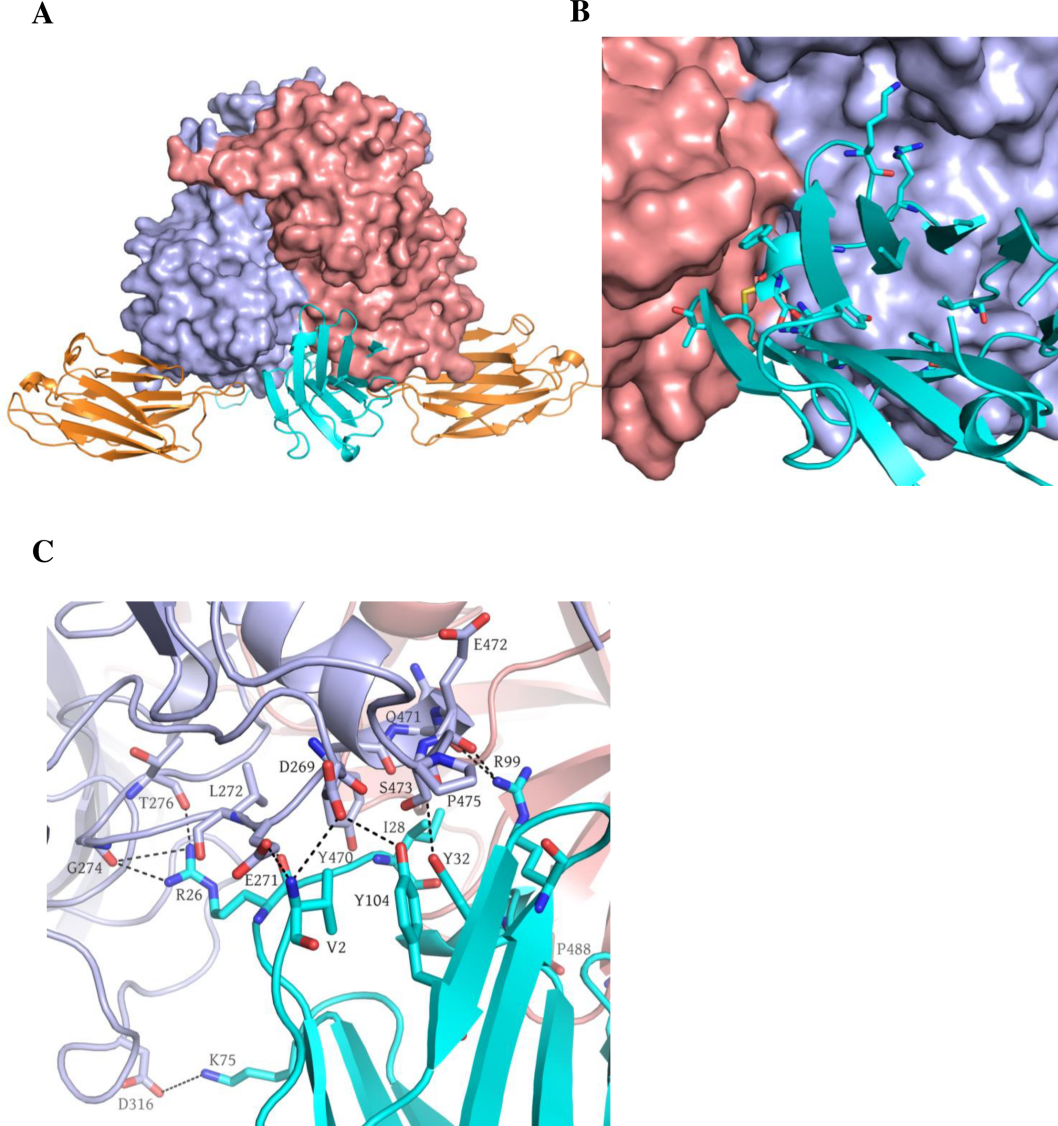
Nano-26 Nano-85 GII.10 P domain double complex structure. GII.10 P domain Nano-26 Nano-85 crystal diffracted to 2.3Å in a C121 space group. Unit cell contained a P domain dimer with two Nano-85 and two Nano-26 molecules. (A) GII.10 P domain is colored as in Fig 3 with Nano-26 (cyan) and Nano-85 (orange). The Nano-85 and Nano-26 binding site in a double complex were identical to binding sites in individual complexes. (B) Nano-26 binds to the cleft between two P domain monomers at the bottom of the P domain dimer. (C) Close up view on the interactions between P domain residues and a Nano-26. Seven direct hydrogen bonds formed between P domain chain B: D269, L272, E274, E471, E472, T276 and Nano-26: V2, R26, R99, and Y104. P domain chain A: I231, P488 and chain B: E271, D316, Y470, and P475 were involved in hydrophobic interactions and two electrostatic interactions with Nano-26: V2, I28, F30, M31, K75, and A102.

Nanobodies were previously shown to aid the crystallization process by increasing protein stability and stabilizing flexible regions [33]. We have already utilized Nano-85 to obtain high-resolution complex structures with three different norovirus P domains [31]. Herein, we used Nano-85 to improve the resolution of the GII.10 P domain Nano-26 complex structure and describe the synchronized binding of two Nanobodies. The initial structure of GII.10 P domain Nano-26 complex was solved to ~3Å resolution. A single crystal of GII.10 P domain Nano-85/Nano-26 double complex diffracted to 2.3Å in C121 space group. Binding epitopes and interactions of both Nanobodies were identical to those in the individual complexes [31]. Nano-26 bound at the bottom of the P domain, perpendicular to Nano-85 binding site (Fig 7A).

Nano-26 binding site comprised of residues from both P domain monomers, although the majority of the P domain interactions involved only one chain (chain B). Nano-26 formed seven direct hydrogen bonds with one P domain monomer (chain B: Asp269, Leu272, Gly274, Gln471, Glu472, and Thr276) (Fig 7B). Both P domain monomers were involved in hydrophobic interactions (chain A: Ile231, Pro488; and chain B: Tyr470 and Pro475) with Nano-26. In addition, two electrostatic interactions contributed to the tight binding. Nano-26 binding residues were mainly conserved between GII genotypes, which correlated well with the broad recognition shown with ELISA (Figs 1 and 4). Although the binding site was distant from the HBGA binding pocket, Nano-26 had a high inhibition capacity in the blocking assay, which also suggested indirect HBGA interference.

### Structure of GII.10 P domain Nano-27 complex

**Fig 8:**
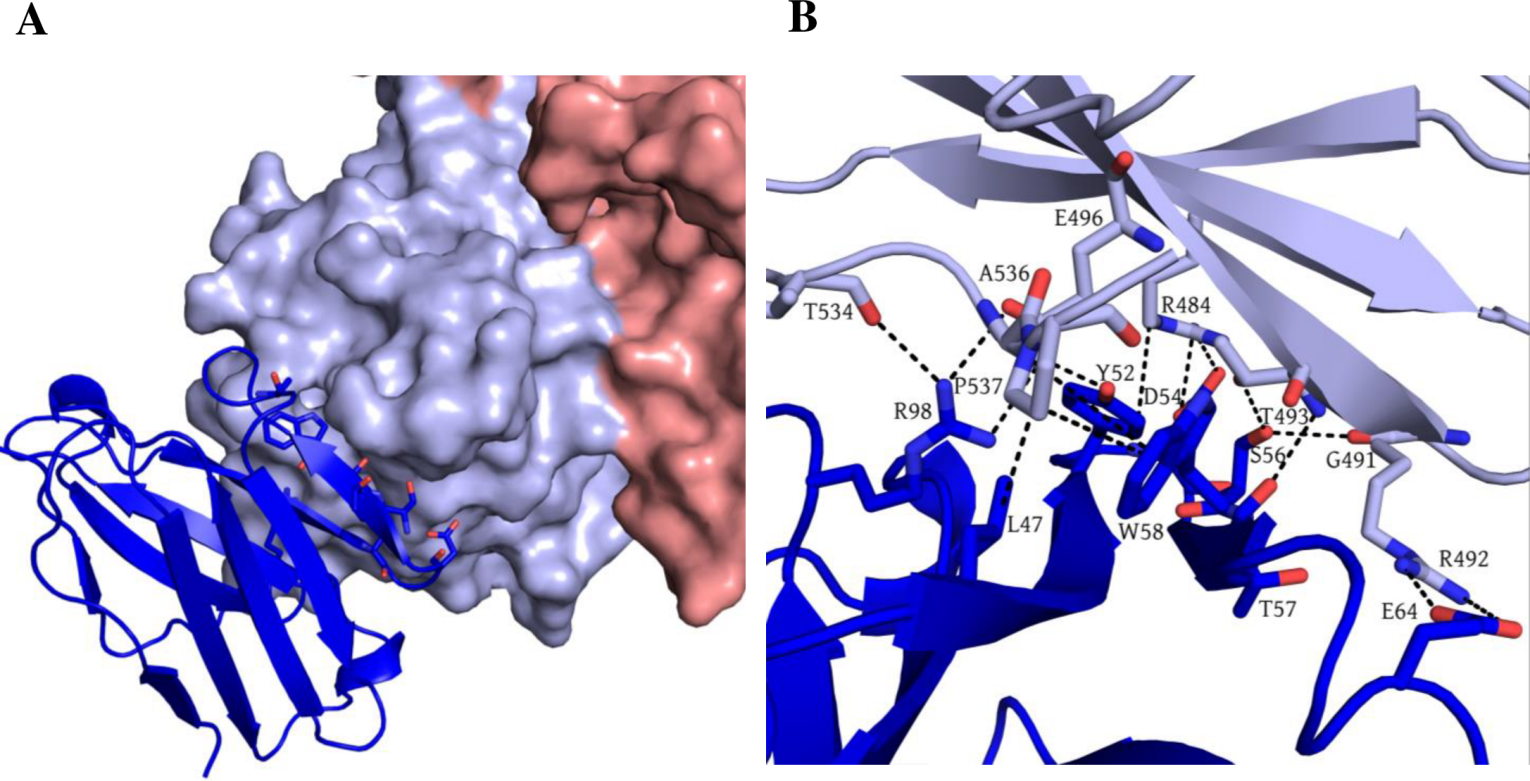
Structure of the GII.10 P domain Nano-27 complex. The X-ray crystal structure of GII.10 P domain Nano-27 complex was solved to 2.9Å resolution. GII.10 P domain is colored according to Fig 3 and Nano-27 (blue). (A) Nano-27 bound to the lower part of P domain monomer. (B) Nano-27 forms an extensive network of hydrogen bonds and hydrophobic interactions with P domain residues. Six P domain residues: R484, G491, R492, T493, E496, and T534 were involved in ten direct hydrogen bonds and two electrostatic interactions with Nano-27: D54, E64, R98, Y52, S56, T57, and E64. Three P domain residues: R484, A536, and P537 were involved in four hydrophobic interactions with Nano-27: W58, Y52, and L47.

The Nano-27 binding epitope was located on the bottom region of the P domain (Fig 8). Interestingly, the binding site partially overlapped the Nano-4 binding site. Six P domain residues (Arg484, Gly491, Arg492, Thr493, Glu496, and Thr534) were involved in ten direct hydrogen bonds and two electrostatic interactions. Three residues (Arg484, Ala536, and Pro537) were involved in four hydrophobic interactions with Nano-27. The Nano-27 binding site comprised six conserved residues and two variable residues. The ELISA data showed that Nano-27 was strain specific, which indicated that certain variable residues likely play a crucial role in cross-reactivity (Fig 4). Similarly to Nano-4, Nano-27 also failed to block VLP binding to HBGAs.

### VLP structural integrity upon Nanobody treatment

**Fig 9:**
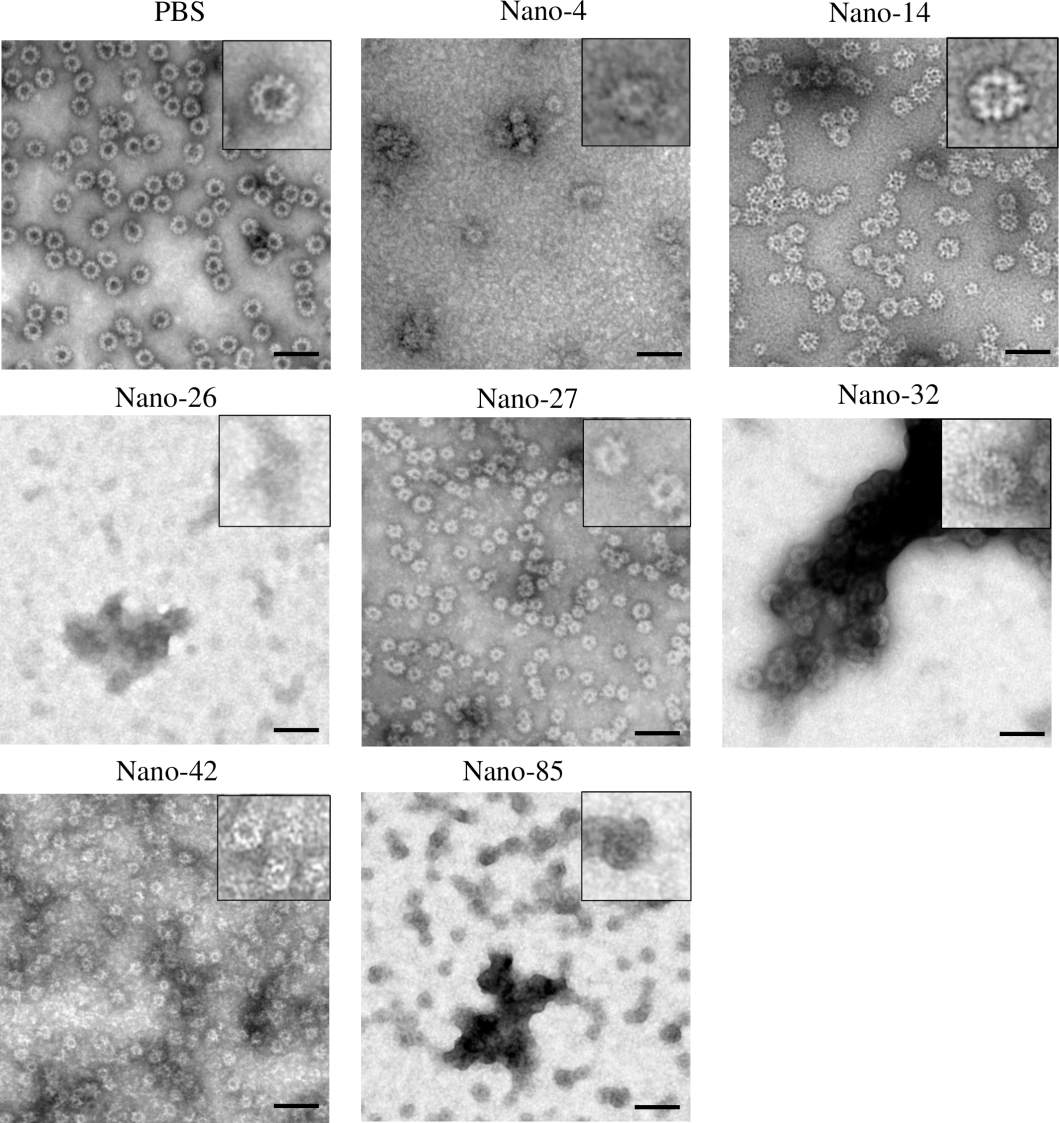
Nanobody treatment of GII.10 norovirus VLPs causes capsid deformation. GII.10 VLPs were treated with each Nanobody for 1 h at room temperature and applied on EM grids for negative staining. Nano-14 treated VLPs preserved the initial morphology, whereas Nano-26, Nano-85 and Nano-42 binding caused changes in particle integrity. Nano-85 treated VLPs were largely broken with a few small-size particles. Nano-4, Nano-27 and Nano-42 treated VLPs tended to shift to the smaller form, whereas Nano-32 treated VLPs formed large aggregates. Negative stain EM images were obtained at 50,000 magnification. The scale bar represents ~50 nm.

We previously showed that Nano-85 was able to disassemble norovirus VLPs [31]. To explore if these six newly identified Nanobodies had a similar ability, we treated native-size VLPs with Nanobodies and examined the treated-particle morphology using EM. Overall, three distinct VLP structural modifications were observed with Nanobody treatment (Fig 9). In the first case, Nano-85 and Nano-26 treatment partially disassembled and deformed the native-size VLPs. Nano-85 treatment also produced a minor fraction of small-size VLPs (20–23 nm). In the second case, Nano-4, and Nano-27 treatment induced a conformational transition from native-size VLPs (35–38 nm) to the small-size VLPs. In case of Nano-42, small and disassembled particles were equally present after treatment. In the third case, Nano-32 treatment produced large aggregates of apparently intact native-size VLPs. None of these effects were observed with Nano-14 treatment.

To investigate a temperature dependence of the Nanobody treatment, we mixed GII.10 VLPs with Nano-85 and Nano-26 at 4°C, room temperature, and 37°C for 30 minutes (S6 Fig). Nano-85 treated VLPs showed a continuous degradation of native-size particles, producing small and/or partially broken particles as major intermediate forms. Nano-26 was more effective across the temperature range and almost completely altered the VLP integrity. The combination of Nano-85 and Nano-26 appeared to cause a more intense degradation of VLPs. The temperature dependence of Nano-85 induced morphological changes indicated the involvement of capsid “breathing” in the disassembly process.

**Fig 10:**
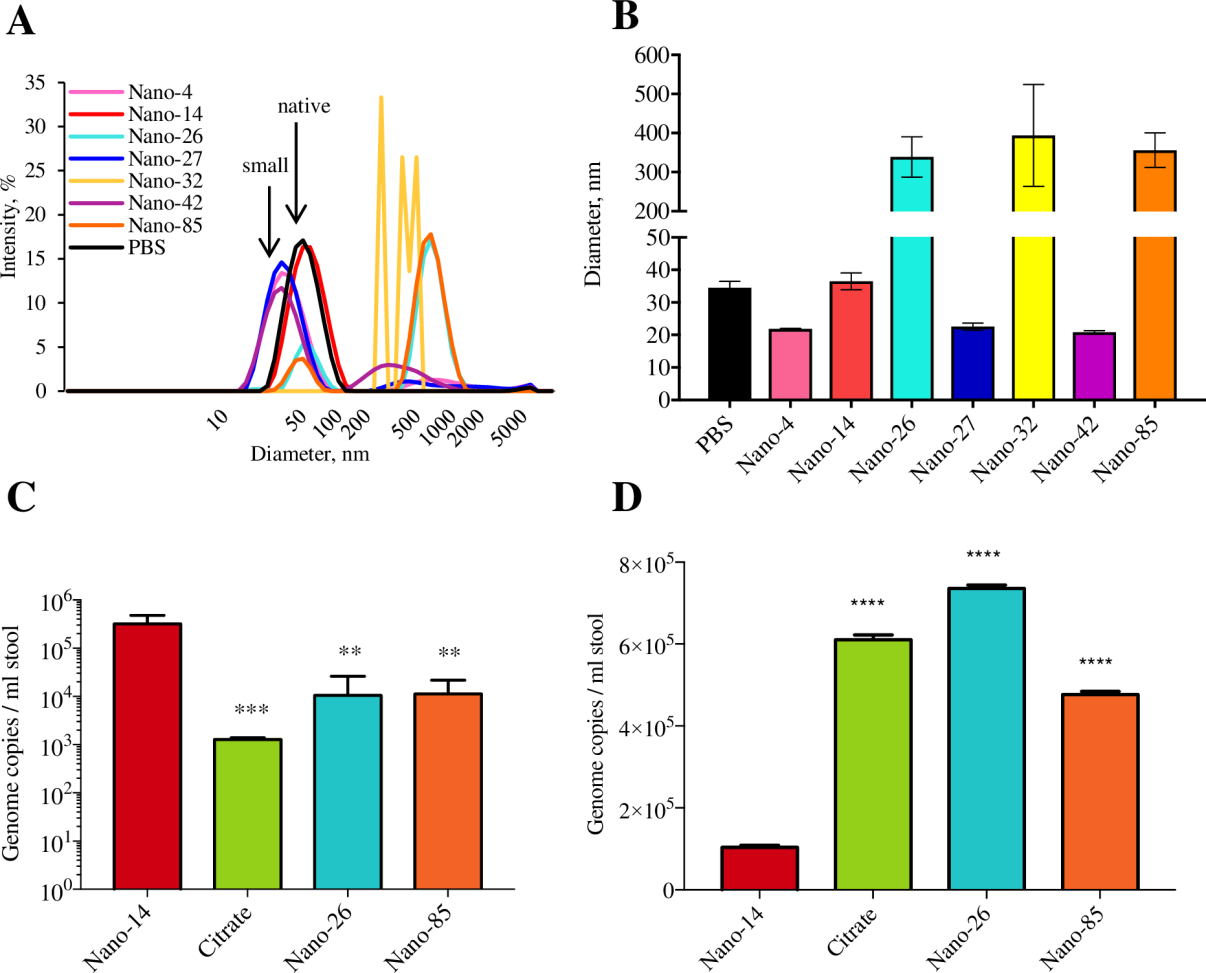
Nanobody treatment leads to changes in norovirus capsid morphology. (A) DLS profiles of Nanobody treated GII.10 VLPs. (B) Average diameters of treated VLPs. Nano-26, Nano-32 and Nano-85 binding caused the formation of large molecular weight aggregates. All experiments were performed in triplicates. (C) Concentrated stool suspension was treated with Nano-14, Nano-26, Nano-85, and 250 mM citrate buffer and subsequently with 50 U of RNAse. Genome copies were quantified with RT-qPCR. Nano-26, Nano-85, and citrate caused a significant decrease in genome copy levels compared to Nano-14. (D) 10% stool suspension was treated with Nanobodies or GHCl and diluted twice with PBS to decrease viral lysis efficiency. Genome copies levels were measured as before and indicated additional lysis in samples pre-treated with Nano-85, Nano-26 and GHCl. Statistical analysis was performed using one-way ANOVA test. Significant differences (P≤0.05) between the treated samples and a negative control (Nano-14 treatment) are marked with stars.

We also performed DLS measurements to quantitatively evaluate GII.10 VLP heterogeneity after Nanobody exposure. Nano-14 treated VLPs had almost identical diameters to native-size particles (37 nm and 35 nm, respectively) (Fig 10A and 10B). Nano-32 treated VLPs displayed 10,000 times increased diameters, confirming the formation of the large aggregates observed using EM. Nano-26 and Nano-85 treated VLPs mainly formed VP1 protein aggregates, although a small peak corresponding to native-size particles remained. Nano-4, Nano-27, and Nano-42 treated VLPs showed peaks corresponding to small-size VLPs (21–23 nm). Overall, the DLS analysis corresponded well with the EM results and provided additional evidence that Nanobody treatment altered the capsid structural integrity.

### Nanobody effects on prevalent GII.4 and GII.17 VLPs

**Fig 11:**
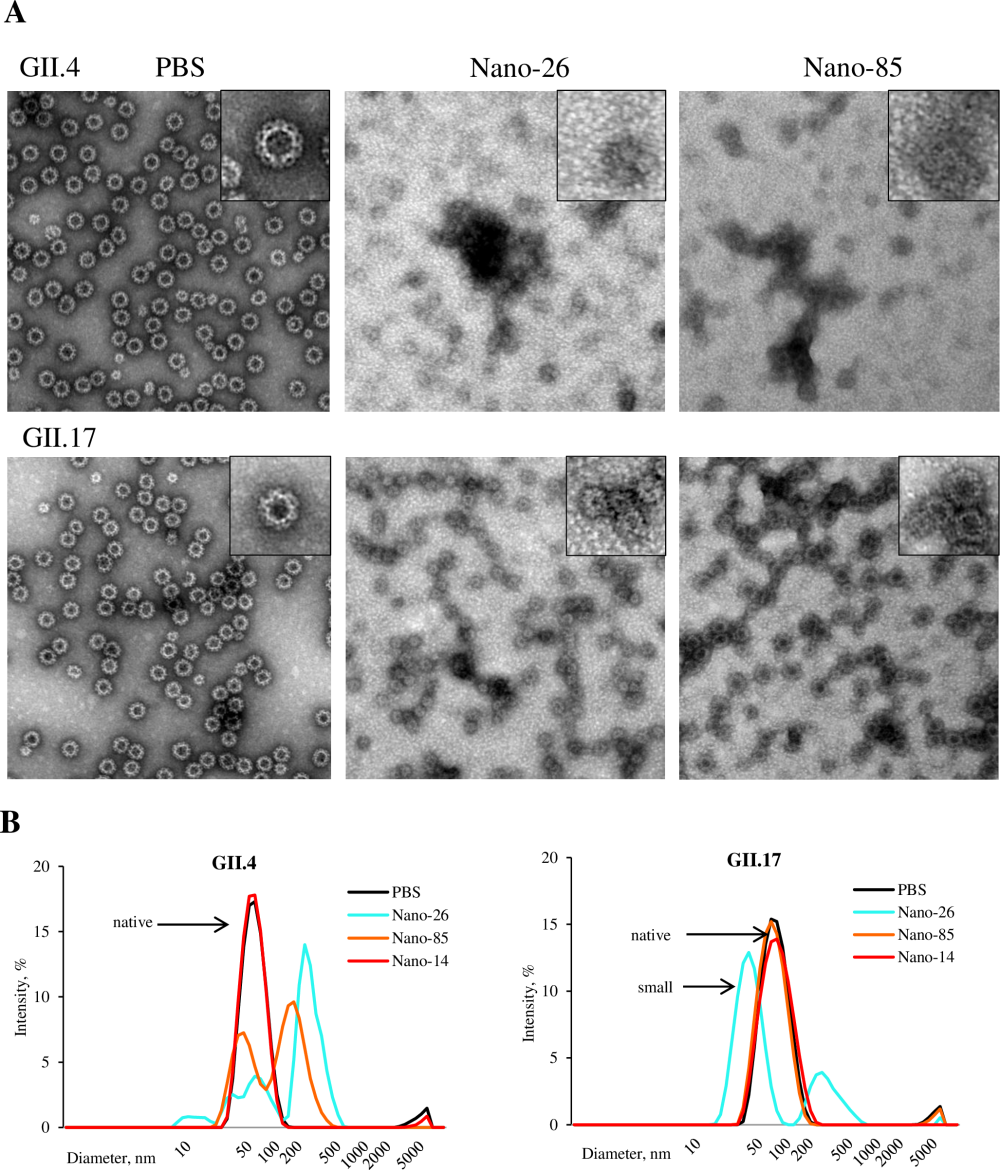
Broadly reactive Nanobodies affect the capsid of prevalent norovirus strains. VLPs of two prevalent norovirus strains GII.4 and GII.17 were incubated with Nano-26 and Nano-85 for 1 h at room temperature. (A) Samples were then applied on EM grids and stained with 1% uranyl acetate. Both Nanobodies degraded GII.4 VLPs. Nano-26 exposure of GII.17 VLPs caused the appearance of VLPs with smaller diameters, whereas Nano-85 seemed to be ineffective, although VLPs appeared to be partially deformed. EM images were obtained at 50,000 magnification, the scale bar represents ~100 nm. (B) DLS profiles of GII.4 2012 and GII.17 VLPs treated with Nano-26 and Nano-85 confirmed the EM observations.

Two Nanobodies, Nano-26 and Nano-85, exhibited broad cross-reactivities coupled with adverse effects on capsid integrity. To understand if these effects were relevant for clinically important norovirus strains, GII.4 (Sydney 2012) and GII.17 VLPs were treated with Nano-26 and Nano-85 (Fig 11). Both Nanobodies lead to malformed and aggregated GII.4 VLPs and produced only a few small-size particles. In the case of GII.17 VLPs, Nano-26 treatment caused the formation of small-size VLPs, whereas Nano-85 seemed to have no notable effect on the particle size. These EM results were supported with the DLS measurements (Fig 11). Overall, these results suggested that effects of the Nanobody treatment might vary among different genotypes.

To further evaluate the binding effects of Nano-26 and Nano-85 on GII.4 (2012) VLPs, we performed time-, temperature-, and concentration-dependent DLS measurements (S7 and S8 Figs). Nano-26 induced changes in the VLP size distribution after 30 seconds, whereas for Nano-85 15 minutes were required to observe the first noticeable effects (S7 Fig). Fluctuations in VLP sizes were more evident at 37°C for both Nano-26 and Nano-85 after 15 minutes incubation (S7A and S7B Fig). Nanobody effects were also concentration dependent, with minimum concentrations of 12.5 μM and 50 μM required for Nano-26 and Nano-85, respectively (S7C and S7D Fig). These results suggested that one Nano-26 molecule per VP1 dimer was sufficient to cause morphological changes, whereas Nano-85 required >2 times molar excess.

### Nanobody effects on GII.4 virions

In order to examine the Nanobody effects on norovirus virions, we implemented a modified RNA exposure assay and viral loads were quantified using real-time RT-PCR. Concentrated GII.4 positive stool samples were treated with the broadly reactive Nano-26 and Nano-85, while Nano-14 was used as a negative control and 250 mM citric buffer was used as a positive control. Treated samples were then subjected to RNAse digestion. Nano-26, Nano-85, and citrate treated stool samples showed reduced genome copy numbers compared to the Nano-14 control (approx. 30 times for Nano-26 and Nano-85 and 250 times for citrate) (Fig 10C). These results suggested that the Nano-26 and Nano-85 opened the virions and released the viral RNA, which was degraded by RNAse. To evaluate the Nanobody effects on norovirus virions more directly, we used a stool sample where RNA degradation was not detected and performed RNA extraction with incomplete lysis step (Fig 10D). Additional degradation caused by Nanobodies or citrate lead to an increased number of genome copies compared to untreated samples. Indeed, Nano-26, Nano-85, and citrate treated samples had higher RNA levels than in the control samples (PBS or Nano-14) (Fig 10D). Although, the fold increase was relatively small (5–7 times), the difference was significant.

To further investigate if Nanobody treatment could render norovirus VLPs vulnerable to proteolytic cleavage, we subjected GII.10, GII.4, and GII.17 VLPs to a 30-minute trypsin digestion after Nanobody exposure and observed the protein bands using SDS-PAGE (S10 Fig). Nano-14 treated VLPs produced similar bands as the untreated VLPs. Nano-26 and Nano-85 treatment resulted in multiple cleavage products for GII.10 and GII.4 VLPs. In the case of GII.17 VLPs, only Nano-26 treatment showed additional cleavage of the capsid protein. Overall, these results suggested that Nano-85 and Nano-26 caused the particles to become structurally unstable, more vulnerable to proteolytic cleavage, and viral RNA exposure.

## Discussion

Structural information of antibody and Nanobody binding sites can be instrumental for understanding the neutralizing and immuno-dominant epitopes as well as motion dynamics of the viral capsid. Numerous neutralizing mAbs have been identified in recent years with diverse neutralization mechanisms [34]. One of the most direct mechanisms is blocking the receptor binding sites. Such neutralizing mAbs and Nanobodies were previously identified for influenza virus, HIV, herpes simplex virus, rhinovirus, and others [35–41]. For example, in the case of HIV, with the aid of an extra long CDR3 loop, the neutralizing Nanobody D7 effectively competed for the CD4 binding site on gp120 protein [42]. Previously described Nanobodies and mAbs with therapeutic potential against human norovirus were also proposed to interfere with the HBGA binding site [20,22–27]. MAb termed NV8812 bound to a conformational epitope on the GI.1 P domain and blocked the binding of norovirus VLPs to human and animal cell lines [24]. Four α-GI mAbs isolated from chimpanzees challenged with norovirus blocked VLP binding to carbohydrates and inhibited hemagglutination, although their precise binding sites were not described [20]. Recently, a GI.1 specific mAb was discovered that sterically hindered the HBGA pocket [22]. In our study, we showed that Nano-14 overlapped with the GII.10 HBGA binding sites and inhibited HBGA binding by steric interference and competition for the pocket. The blocking abilities of Nano-14 were also comparable to previously reported blocking Nanobodies (IC_50_ = 0.34–2.0 μg/ml) [23], scFv fragments (IC_50_ = 0.3–1.5 μg/ml) [43], and mAbs (IC_50_ = 0.12–0.74) μg/ml [20,28]. Although exhibiting high inhibition capacity, these mAbs and Nanobodies tend to be strain specific.

The use of mAbs or Nanobodies directed to the HBGA pocket may inherently suffer from the variations and constantly changing amino acids in this region. Therefore, there is a need to identify additional neutralization epitopes, which are less susceptible to sequence variations. Indeed, Nano-32 recognition epitope was distant from the HBGA binding pocket and blocked VLP binding to HBGAs. A similar phenomenon was previously discussed with the norovirus specific blockade mAb NVB71.4, where neither particle disassembly nor steric hindrance could explain NVB71.4 blockade activity [25]. However, it was suggested that the NERK motif (residues 310, 316, 484, and 493 according to GII.4 numbering) could function as a conformational regulator through an allosteric effect [25]. Interestingly, two of these residues were directly involved in Nano-32 binding, suggesting a similar blockage mechanism as observed with mAb NVB71.4. Nano-32 induced conformational rearrangement of several P domain loops, which in turn altered the hydrophobic landscape of the P domain surface. This rearrangement likely caused the particle aggregation leading to interference at the HBGA binding pocket. An inhibition mechanism by allosteric interference was previously described for highly neutralizing mAbs against HIV and dengue virus [44,45]. Also, a recent study showed that the PGT121 mAbs against HIV gp121 protein inhibited CD4 binding, although the binding epitope was remote from the CD4 binding site. Moreover, dengue virus neutralizing mAb 1A1D-2 bound to a partially occluded epitope on envelope glycoprotein E and promoted particle reorganization [45]. These changes in viral surface were likely responsible for the inhibitory properties by this mAb.

To allow structural rearrangement to occur during viral entry and uncoating, the viral capsid proteins need to be exceptionally dynamic. Internal plasticity and motions of the capsid proteins can allow access to buried regions, which often play an important role in the viral life cycle [46]. Indeed, multiple antibodies against picornaviruses and flaviviruses that bind to normally inaccessible sites on the viral capsid were shown to be highly neutralizing [47–52]. For rhinoviruses and polioviruses, buried regions of internal VP4 protein are transiently exposed due to the capsid “breathing” and are targeted by neutralizing mAbs. These cryptic epitopes are often very conserved and therefore provide cross-serotypic neutralization. We previously identified a broadly reactive norovirus mAb [53] and Nano-85 that bound to a conserved region that was occluded in the context of native-size particles [31]. Here, we identified four novel Nanobodies (Nano-4, Nano-26, Nano-42, and Nano-27) that bound to the similar internal and poorly accessible epitopes as Nano-85. In comparison with Nano-85, the binding sites of Nano-27 and Nano-4 were located closer to the P domain crown. In context of the complete particle, this position had fewer steric clashes with neighboring P domains. The Nano-26 epitope was located at the bottom of the P domain, albeit perpendicular to Nano-85 binding site. Although completely different, Nano-26 recognition epitope was also conserved and poorly accessible (Fig 7). The time- and temperature-dependence of the Nanobody-induced degradation suggested an important role of conformational mobility and capsid “breathing” in Nano-85 and Nano-26 binding to these hidden epitopes [46].

**Fig 12:**
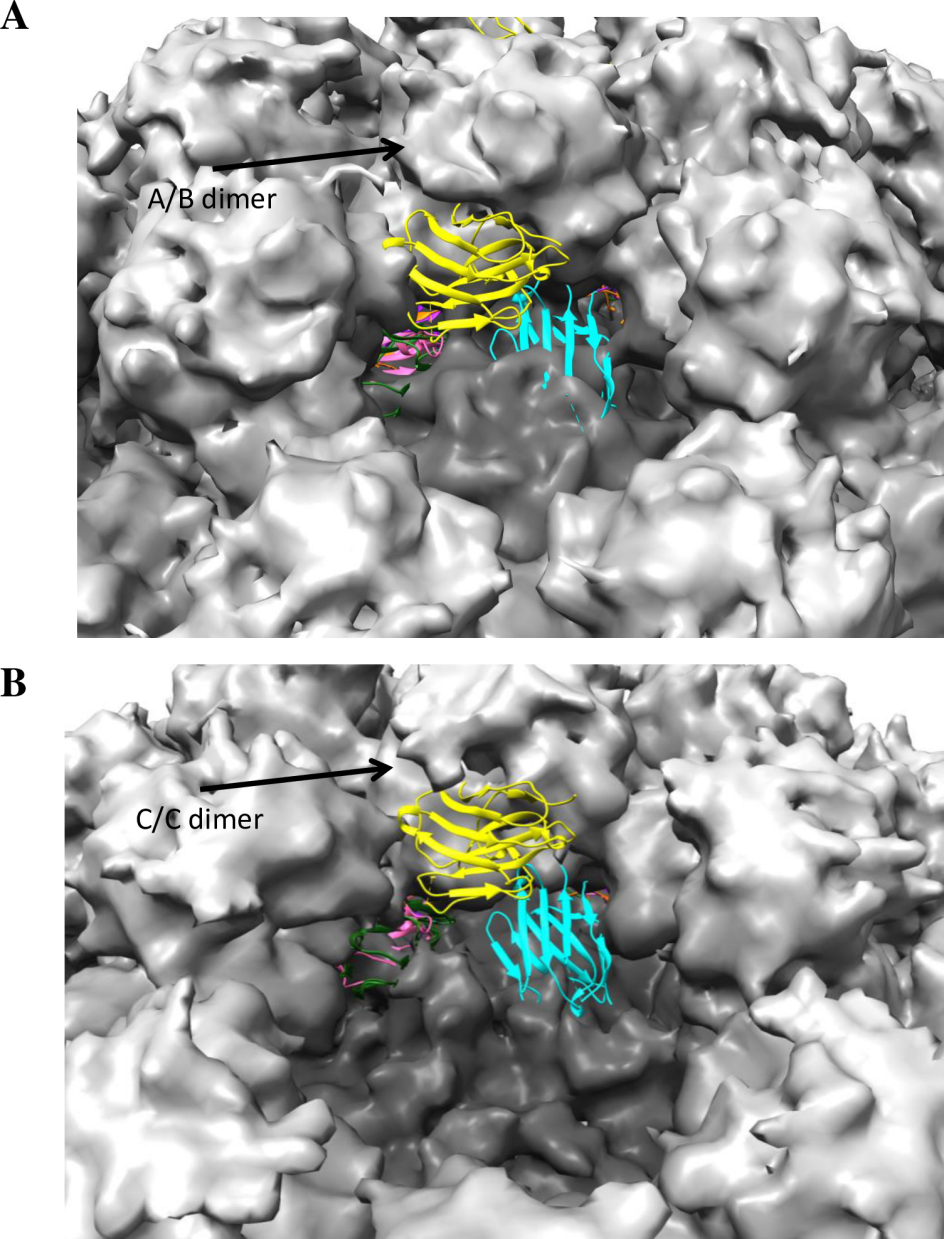
Nanobody binding in context of the whole particle. Nanobodies GII.10 P domain complex structures were superimposed with A/B dimer (A) or C/C dimer (B) of the GII.10 VLP cryo-EM structure. A view from 5-fold axes (A) or 3-fold axis is presented (B). Two Nanobodies bound to the relatively more exposed sites, Nano-26 (cyan) and Nano-32 (yellow). Nano-4 (hot pink), Nano-14 (red), Nano-25 (dark green), Nano-27 (blue), Nano-42 (dark purple), and Nano-85 (orange) bound to occluded epitopes on the bottom of the P domain and were poorly visible in context of the whole particle.

Due to their small size and high affinities, the rapid binding of the Nanobodies provided a means to trap transiently exposed regions, otherwise buried in the native state of the particles. Trapping the particles in a particular conformation or otherwise inhibiting capsid “breathing” is a common antiviral strategy shared by many neutralizing mAbs, Nanobodies, and drugs against HIV, flaviviruses, picornaviruses, influenza, and others [54–60]. For example, several neutralizing Nanobodies against poliovirus and respiratory syncytial virus were shown to specifically stabilize either the native or expanded conformation of capsid, preventing it from further rearrangement necessary for the infection process [30,61]. It is plausible that in the case of norovirus Nanobodies described here, binding resulted in a stabilization of the particular P domain conformation, thus reducing the mobility and influencing the position on the S domain. The interaction between S and P domains was previously shown to control the size and stability of the GI.1 norovirus capsid [62]. Superposition of P domain Nano-26 complex on the cryo-EM VLP structure revealed an extensive clash with the S domain (Fig 12). Nano-26 binding likely disrupted normal S-P domain orientations, which consequently resulted in particle disassembly. Nano-26 required less time and concentration to achieve particle disassembly than Nano-85. This observation suggested that the restriction of a normal S-P domain relationship had a more destabilizing effect than interference with P-P domain interactions. Of note, only Nano-26 was able to influence the morphology of GII.17 VLPs, whereas GII.17 VLPs tolerated Nano-85 binding. Apparently, Nano-26 binding stabilized the S-P domain conformation that was incompatible with the morphology of native-size GII.17 particles, but supported the formation of small-size VLPs. Furthermore, three other Nanobodies, Nano-27, Nano-4 and Nano-42, drove a shift from native-size GII.10 VLPs to a smaller-size form. Likely, these Nanobodies could selectively stabilize the A/B conformation of the P dimer. The inability of the A/B dimer to reassemble into C/C dimers could lead to the formation of small particles, where all dimers are identical and resemble A/B dimer for T = 3 capsids.

Interference with the capsid motions and integrity provides one possible explanation for the blocking properties of both Nano-26 and Nano-85 in the surrogate neutralization assay. Nanobody binding caused the loss of normal VLP morphology and the treated VLPs showed a reduced signal in the blocking assays. Indeed, chemically disassembled VLPs showed no binding in a PGM assay (S10 Fig). These observations support the assumption that Nano-85 and Nano-26 inhibited the binding of norovirus VLPs to HBGAs by compromising capsid morphology instead of directly competing for the HBGA pocket. Interestingly, Nano-42, Nano-27, and Nano-4, which stimulate the formation of small-size particles, did not interfere with the attachment to the HBGAs. It was previously shown that small-size VLPs effectively bound to the surface of CaCo2 cells and competed with the native-size VLPs [24]. Apparently, the small-size VLPs that resulted from Nanobody exposure were equally able to bind HBGAs.

Intriguingly, our structural data indicated that closely overlapping epitopes are responsible for distinct functions. A striking example is Nano-42 and Nano-85, which despite having almost identical binding footprints, showed distinct binding and blocking properties. Nano-42 seemed to be less effective in disassembling the VLPs compared to Nano-85. Similar observations were previously reported for 80S poliovirus specific Nanobodies, where despite identical binding sites, the structures of the expanded virus differed in each complex [48]. Likewise, although Nano-4 and Nano-27 shared five of eight binding residues, Nano-27 was strain specific, whereas Nano-4 was cross-reactive. Even though the epitopes closely overlap with Nano-85 binding site, these Nanobodies did not exhibit blocking properties. Analysis of GII.10 P domain residues involved in Nano-27, Nano-4, and Nano-42 binding suggested that residues 484, 491–493, and 496 might constitute the molecular switch responsible for preferential assembly of small particles. Thus, additional high-resolution structural information could be instrumental in understanding epitope-function relationships by providing the exact location and interactions of the binding partners. This information might remain elusive when more general epitope mapping methods are used.

In addition to identification of functional epitopes on the norovirus capsid, our data provided insights of Nanobody potential neutralization properties in context of infectious norovirus virions. Recently, it was shown that silver dihydrogen citrate exposure compromises GII.4 VLPs integrity and facilitates viral RNA degradation [63]. Similarly, we showed that Nanobody-induced morphological changes of norovirus capsid resulted in exposure of viral RNA from the norovirus virions in clinical samples. The naked RNA was especially vulnerable to RNAse digestion and a similar RNA degradation assay was shown to greatly reduce the infectivity of murine norovirus [64]. In addition to exposing the viral RNA, Nanobodies increased the susceptibility of capsid protein to proteases, which are abundant in the gut. Although the exact role of proteolytic cleavage in the norovirus life cycle is largely unknown, cleaved capsid protein was shown to lose the ability to bind HBGA and maintain capsid assembly [65].

In summary, we identified several Nanobodies that impaired normal capsid motions, assembly, and integrity with subsequent release of viral RNA. Four Nanobodies blocked norovirus binding to cell attachment factors (HBGAs), utilizing three distinct inhibition mechanisms: steric occlusion of the HBGA binding site, allosteric interference, and violation of normal capsid morphology. Therefore, Nanobodies could act as broad inhibitors in multiple stages of the norovirus life cycle. The Nanobody capacity to inhibit human norovirus infections in the recently developed cell culture needs to be further evaluated. Nevertheless, the extensive evidence that interference with viral capsid dynamics could impair normal functioning suggested that Nanobodies could become effective norovirus therapeutics in future.

## Materials and methods

### P domain production

The norovirus P domains, GI.1 (Norwalk virus, Genbank accession number M87661), GI.11 (Akabane, EF547396), GII.1 (Hawaii, U07611), GII.2 (Snow Mountain, AY134748), GII.4 (Sydney-2012, JX459908 and Saga4 2006, AB447457), GII.10 (Vietnam026, AF504671), GII.12 (Hiro, AB044366), and GII.17 (Kawasaki308, LC037415 were expressed in *E*.*coli*, purified and stored in GFB (25mM Tris-HCl pH7.6, 0.3M NaCl) [66]. The full-length capsid genes, GI.1 (AY502016.1), GI.11, GII.1, GII.2, GII.4, GII.10, GII.12, and GII.17, were expressed in insect cells using the baculovirus expression system and stored in PBS [67,68].

### Generation of norovirus specific Nanobodies

Norovirus specific Nanobodies were produced at VIB Nanobody service facility, Belgium as previously described [31]. Briefly, a single alpaca was injected with GII.10 VLPs. A VHH library was constructed from isolated peripheral blood lymphocytes and screened for the presence of antigen-specific Nanobodies using phage display. Thirty-five Nanobodies were isolated and allocated to 17 distinct groups based on a sequence alignment. Six Nanobodies (Nano-4, Nano-14, Nano-26, Nano-32, Nano-42, Nano-27, and Nano-8) that represented different groups were analyzed in this study. The Nanobody genes were cloned to pHEN6C vector, expressed in WK6 *E*.*coli* cells, purified and stored in PBS or GFB.

### Direct antigen ELISA

Nanobody titers to norovirus P domains or VLPs were quantified with direct ELISA (17). Briefly, microtiter plates were coated with 7 μg/ml of GII.10 P domains or 2 μg/ml of GII.10 VLPs. For cross-reaction experiments, 15 μg/ml P domain and 4 μg/ml VLPs were coated on ELISA plates. The VLPs or P domain were detected with serially diluted Nanobodies and HRP-conjugated mouse α-His-tag monoclonal antibody. Absorbance was measured at 490 nm (OD_490_) and all experiments were performed in triplicate.

### Blocking assays

Pig gastric mucin (PGM) and saliva blocking assays were performed as previously described [69]. Briefly, ELISA plates were coated with 10 μg/ml PGM (Sigma, Germany) or with saliva type A or B diluted in PBS 1:2000. Nanobodies were two-fold serially diluted in PBS containing 2.5 μg/ml GII.10 VLPs (for PGM assay), 0.5 μg/ml GII.10 VLPs (for saliva assay) or 0.5 μg/ml GII.4 2006 VLPs (both PGM and saliva assay) and incubated for 1 h at RT. The VLPs-Nanobodies mixture was added to the plates and bound VLPs were detected with a α-GII.10 or α-GII.4 VLPs rabbit polyclonal antibody. For synthetic HBGA blocking assay, 10 μg/ml synthetic blood type B trisaccharide amine derivative (Dextra, UK) was coated on Pierce maleic anhydride activated plates (Thermo Fisher Scientific) overnight at 4C. Serially diluted Nanobodies were pre-incubated with 5 μg/ml GII.4 VLPs for 1h at RT. Following steps were performed as above. The binding of VLPs-only was set as a reference value corresponding to a 100% binding. The half maximal inhibitory concentrations (IC_50_) values for Nanobody inhibition were calculated using GraphPad Prism (6.0a).

### Isothermal Calorimetry measurements

Isothermal Calorimetry **(**ITC) experiments were performed using an ITC-200 (Malvern, UK). Samples were dialyzed into the identical buffer (GFB or PBS) and filtered prior titration experiments. Titrations were performed at 25°C by injecting consecutive (1–2 μl) aliquots of Nanobodies (100–150 μM) into P domain (10–20 μM) with 150 second intervals. The binding data was corrected for the heat of dilution and fit to a one-site binding model to calculate the equilibrium binding constant, K_A_, and the binding parameters, N and ΔH. Binding sites were assumed to be identical. For the competitive ITC measurements, the P domain was mixed with Nano-4, Nano-42, and Nano-27 in a 1:1 molar ratio. Titrations with Nano-85 were then performed as above.

### P domain and Nanobody complex purification and crystallization

P domain and Nanobody complexes were purified by size exclusion chromatography (39). The P domain and Nanobody complexes were crystallized using the following conditions: GII.10 P domain Nano-26/Nano-85 [0.1 M sodium citrate, 40% (w/v) PEG600]; GII.17 P domain Nano-4 [0.2 M calcium acetate, 10% (w/v) PEG8000, 0.1 M imidazole (pH 6.5)]; GII.10 P domain Nano-42 [0.2 M potassium iodide, 20% (w/v) PEG3350]; GII.10 P domain Nano-14 [0.1 M sodium citrate (pH 5.5), 20% (w/v) PEG3000]; GII.10 P domain Nano-32 [0.2 M magnesium formate]; and GII.10 P domain Nano-27 [2 M sodium chloride, 0.1 M sodium acetate]. Crystals were grown in a 1:1 mixture of the protein sample and mother liquor at 18°C. Prior to data collection, crystals were transferred to a cryoprotectant containing the mother liquor in 30% ethylene glycol, followed by flash freezing in liquid nitrogen.

### Data collection, structure solution, and refinement

X-ray diffraction data were collected at the European Synchrotron Radiation Facility, France at beamline BM30, ID30A, ID23-1 A and processed with XDS [70]. Structures were solved by molecular replacement in PHASER *Phaser-MR* [71] using GII.10 P domain (PDB ID 3ONU) or GII.17 P domain (5F4M) and a Nano-85 (4X7D) as search models. Structures were refined in multiple rounds of manual model building in COOT [72] and refined with PHENIX [73]. Alternative binding interfaces derived from the crystal packing were analyzed using an online server PDBePISA. The orientation of the Nanobody with the highest interface surface area and contact with CDRs was selected as the biologically relevant interface. Atomic coordinates were deposited to the Protein Data Bank (PDB).

### Electron microscopy and dynamic light scattering

The norovirus VLP morphology was analyzed using negative stain electron microscopy (EM) as previously described [31]. Nanobodies (1 mg/ml) and VLPs (1 mg/ml) were mixed in 1:1 ratio and incubated for 1 h at room temperature. Prior to loading on carbon coated EM grids, all samples were diluted 30 times with distilled water. Grids were washed two times with distilled water and stained with 1% uranyl acetate. The grids were examined on a Zeiss 910 electron microscope (Zeiss, Oberhofen, Germany) at 50,000-fold magnification. VLP diameter was measured with ImageJ software using calibrated pixel/nm scale bar. The hydrodynamic diameters of treated and untreated norovirus VLPs were measured using dynamic light scattering (DLS) on ZetaSizer Nano (Malvern Instruments, UK). Samples were diluted 1:50 with PBS up to a final volume of 1 ml. Three × 12 measurement runs were performed with standard settings (Refractive Index 1.331, viscosity 0.89, temperature 25°C). The average result was created with ZetaSizer software.

### Stool treatment and real-time PCR

In order to determine the effects of the Nanobodies on native virions, we collected GII.4 positive stool samples from two individuals with acute norovirus infection [74]. A 10% (w/v) stool suspension was prepared in PBS and clarified by centrifugation at 10,000 × g for 10 min. First stool sample was concentrated by ultracentrifugation at 285,000 × g for 3 h at 4°C. Then, 70 μl of the supernatant were treated with 150 μl of each Nanobody (1 mg/ml) for 30 min at room temperature. Samples were digested with 50 U of RNAse One (Promega, Germany) for 30 min at 37°C. After treatment total RNA was extracted with QIAamp Viral RNA extraction kit (Qiagen, Hilden, Germany). One step RT-qPCR was performed with previously published GII.4 primers NKP2F (5’-ATGTTYAGRTGGATGAGATTCTC-3’), NK2R (5’-TCGACGCCATCTTCATTCAC-3’) and probe RING2-TP (5’-FAM-TGGGAG GGCGATCGCAATCT-TAMRA-3’) using qScript XLT One-Step RT-qPCR ToughMix (Quantabio, USA). For incomplete lysis, samples were diluted twice with PBS prior to RNA extraction with shortened incubation time. cDNA was synthesized using High Capacity cDNA Reverse Transcription Kit (Applied Biosystems, Foster City, USA). qPCR with melt curve analysis was performed using SYBR Green Master Mix (Bio-Rad, Hercules, USA). GII.4 specific primers, sense JJV2F (5’-CAAGAGTCAATGTTTAGGTGGATGAG-3’) and antisense COG2R (5’- TCGACGCCATCTTCATTCACA-3’) were used for norovirus detection as previously described [63]. Viral load was quantified by comparison to a standard curve of GII.4 norovirus RNA transcripts of a known concentration. Average values for two independent experiments for concentrated virus and three independent experiments for RNAse free stool are presented. Statistical analysis was performed using one-way ANOVA test. Differences were considered significant when P≤0.05.

### Trypsin digestion

To evaluate the impact of Nanobody binding on capsid susceptibility to proteolytic digestion norovirus VLPs (1 mg/ml) were incubated with Nanobodies (1 mg/ml) in 1:1 ratio for 30 min at 37°C. Then, trypsin-EDTA was added to final concentration of 10 μg/ml for 30 min at 37°C. The concentration of trypsin was chosen to yield only partial cleavage with visible intermediate products. After digestion, samples were loaded on the SDS-12% polyacrylamide gel and stained with coomassie stain.

## Supporting information

S1 Fig Nanobody binding to GII.10 VLPs and P domain and cross-reactivity. Nanobody binding characteristics were analyzed using GII.10 VLPs and P domain in a direct ELISA. Plates were coated with (A) GII.10 VLPs, (B) GII.10 P domain. Nano-42, Nano-14, Nano-26 were the strongest binders and detected GII.10 VLPs at a dilution of ~50 ng/ml. Nano-4 detected VLPs at a lower dilution of 0.1 μg/ml. Nano-32 detected VLPs at concentrations above 0.4 μg/ml and Nano-27 above 1.5 μg/ml. A similar binding pattern was observed with the GII.10 P domain, where Nano-42, Nano-4, Nano-14, Nano-26 detected P domain in concentrations up to 20 ng/ml. Nano-32, and Nano-27 reacted with the P domain at concentrations above 0.2 μg/ml. and 1.6 μg/ml respectively. (C-E) Nano-14, Nano-27 and Nano-32 bound only GII.10 P domain and showed no cross-reactivity to any other GII P domains (15 μg/ml) or to GI.1 and GI.11 VLPs (4 μg/ml).
(TIF)

S2 Fig Saliva and HBGA blocking assays. Saliva blocking assay with GII.10 VLPs (2.5 μg/ml) was performed similarly to PGM binding assay. (A) Nano-14, Nano-26, and Nano-32 inhibited 50% of the binding (IC_50_) to A type saliva at 0.4, 2.6, and 3.1 μg/ml, respectively. (B) For B type saliva IC_50_ values for Nano-14, Nano-26, and Nano-32 were 1.1, 4.3, and 1.8 μg/ml, respectively. Nano-85 showed only weak blocking potential. Binding was expressed as a percentage of the untreated VLP binding (100%). (C) Inhibition of GII.4 VLPs (0.5 μg/ml) binding to synthetic B-tri saccharide. Both Nano-26 and Nano-85 showed a complete inhibition at 10 μg/ml and no inhibition at 1 μg/ml. (D) Inhibition of GII.4 VLPs (0.5 μg/ml) binding to synthetic B type saliva. Nano-26 and Nano-85 blocked GII.4 VLP binding with IC50 of 0.7 and 1.2 μg/ml. All experiments were performed in triplicate (error bars are shown) and the cutoff was set at an OD_490_ of 0.15 (dashed line).
(TIF)

S3 Fig Thermodynamic properties of Nanobody binding to P domain. Titrations were performed at 25°C by injecting consecutive aliquots of 100–150 μM Nanobodies into 10–20 μM GII.10 P domain P domain. Examples of the titrations (upper panels) are shown. The binding isotherm was calculated using a single binding site model after subtraction of the heat of dilution (lower panels). Nano-32 binding to the P domain exhibited endothermic type of reaction, whereas all other Nanobodies showed exothermic binding reaction.
(TIF)

S4 Fig Competitive thermodynamic properties of Nanobody binding to P domain. For the competitive ITC measurements, the P domain was pre-mixed with Nano-4, Nano-14, Nano-26, Nano-27, and Nano-42 in a 1:1 molar ratio. Standard titrations with Nano-85 were then performed. Titrations were done at 25°C by injecting consecutive aliquots of 100 μM Nanobody into 15 μM GII.10 P domain P domain. Examples of the titrations (upper panels) are shown. (A-C) Titration to P domain Nano-4, Nano-27 and Nano-42 showed the absence of heat release associated with injections, indicating the lack of binding. (D, E) Nano-85 showed the binding to GII.10 P domain Nano-26 and Nano-14 complexes with exothermic type of reaction, which resembled the binding of Nano-85 to P domain alone. The binding isotherm was calculated using a single binding site model after subtraction of the heat of dilution (lower panels).
(TIF)

S5 Fig Conformational changes in GII.10 P domain upon Nano-32 binding. Several loops of GII.10 P domain in complex with Nano-32 had altered conformation compared to unliganded P domain. Loop between residues 295–300 was positioned symmetrically in both monomers which was not observed in apo-structure, but was characteristic for GII.10 P domain in complex with 30 mM B-tri saccharide (PDB code 4Z4Z). Loop 343–352 was partially disordered and deviated 4.3Å away from its position in unliganded structure. Loops 309–314, 418–420 as well as 487–491, 517–522 had slightly shifted conformation (2-3Å).
(TIF)

S6 Fig VLPs exposed to Nanobodies exhibit altered morphology. GII.10 VLPs were pre-incubated with Nano-85, Nano-26, or with both Nano-85 and Nano-26 for 30 min at 4°C, room temperature, and 37°C. After treatment VLPs were subjected to negative staining and examined by EM at 50,000 magnification. VLPs exposed to Nano-85 showed a temperature dependence of morphological changes. At 4°C a large portion of native 35–37 nm VLPs were visible, whereas at RT small 20–23 nm VLPs appeared and prevailed at 37°C. In case of Nano-26 and joint Nano-26 and Nano-85 treatment VLPs were largely degraded at any tested temperature.
(TIF)

S7 Fig Time course of Nanobody induced capsid heterogeneity. GII.4 VLPs were incubated with Nano-26 (A) or Nano-85 (B) for indicated periods of time at room temperature and DLS profiles were then measured. Peaks corresponding to large molecular weight aggregates appeared after 30 seconds in case of Nano-26 treated VLPs and 15 min for Nano-85. Arrows indicate native size VLPs (35–37 nm in diameter) and small VLPs (20–23 nm).
(TIF)

S8 Fig Temperature and concentration dependence of GII.4 VLP size distribution after Nanobody treatment. Hydrodynamic diameters of GII.4 VLPs treated with Nano-26 (A) or Nano-85 (B) at 4°C, RT, 37°C for 15 min were measured using DLS. VLPs had an increased particle heterogeneity at 37°C compared to 4°C and RT. Additionally, VLPs were pre-incubated with different concentrations of Nano-26 (C) or Nano-85 (D) for one hour at RT. Size distribution was altered at concentrations over 12.5 μM for Nano-26 and 50 μM for Nano-85.
(TIF)

S9 Fig Proteolytic digestion of norovirus VLPs. GII.10, GII.4, and GII.17 VLPs (1 mg/ml) were treated with 1 mg/ml of Nano-14 (lane N14), Nano-26 (lane N26), Nano-85 (lane N85), or PBS for 30 min at 37C. After the treatment samples were exposed to typsin (10 μg/ml final concentration) for additional 30 min at 37C. Samples were then run on the SDS PAGE and stained with Coomassie. VLPs without trypsin cleavage correspond to the last lane. Nano-14 does not impact the rate of protease degradation, whereas Nano-26 and Nano-85 significantly increased the digestion efficiency.
(TIF)

S10 Fig PGM binding assay with norovirus VLPs. GII.10, GII.4, and GII.17 VLPs (A, B and C, respectively) were serially diluted in PBS, or 1M Tris buffer (pH 10), and incubated at room temperature for one or ten hours. Standard PGM binding assay was then performed using polyclonal serum against GII.10 for detection of GII.10 VLPs and polyclonal serum against GII.4 for detection of GII.4 and GII.17 VLPs. VLPs disassembled with pH 10 for ten hours showed no binding to PGM. Incubation with pH 10 for one hour greatly reduced the binding of GII.10 VLPs and completely abolished the binding of GII.4 and GII.17 VLPs.
(TIF)
