# Supplementary figures and images for: Nanobodies targeting norovirus capsid reveal functional epitopes and potential mechanisms of neutralization
Source: PLoS Pathog. 2017 Nov 2;13(11):e1006636. doi: 10.1371/journal.ppat.1006636 (PMC5667739; doi:10.1371/journal.ppat.1006636)

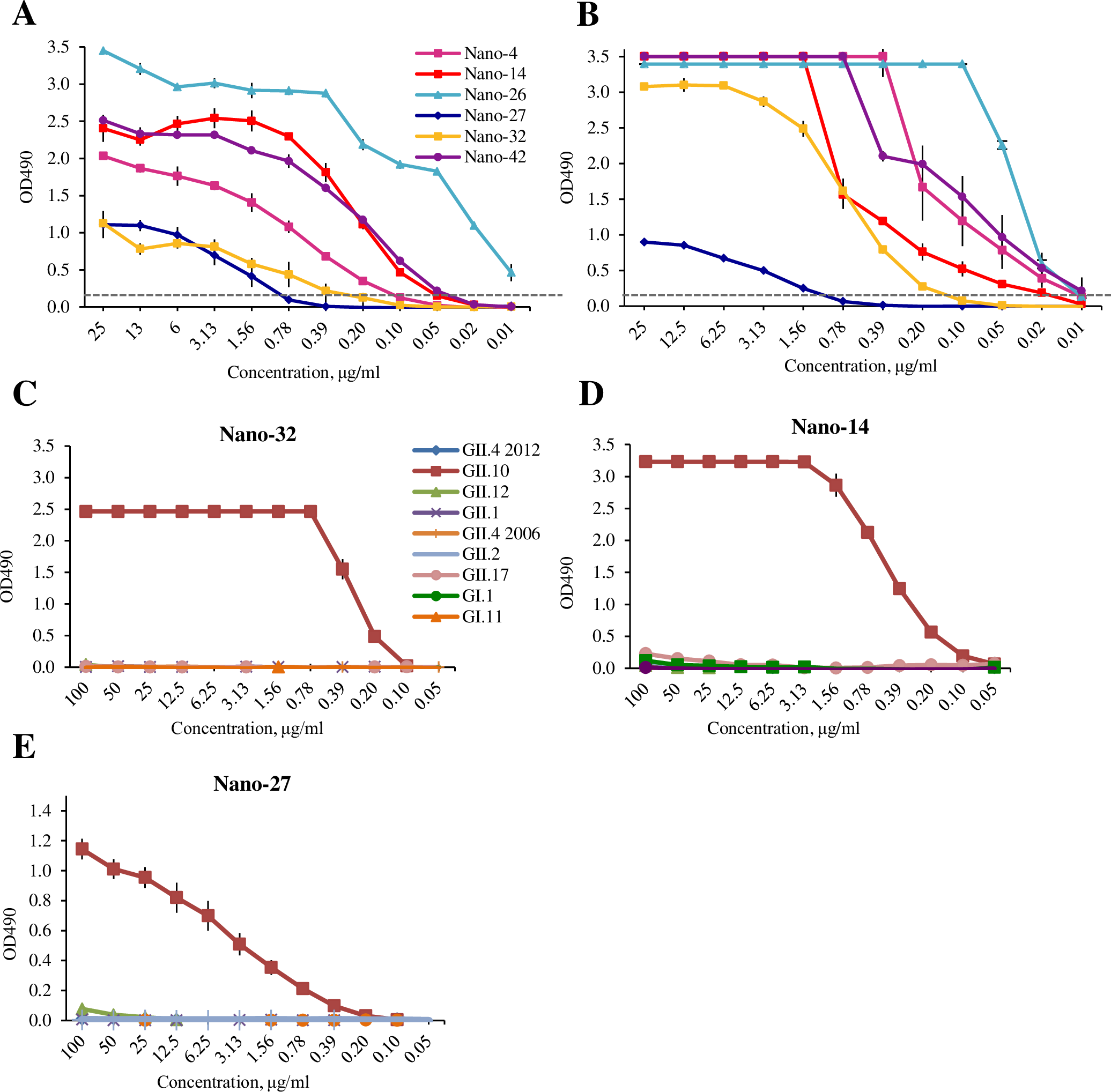

Supplement: S1 Fig — Nanobody binding characteristics were analyzed using GII.10 VLPs and P domain in a direct ELISA. Plates were coated with (A) GII.10 VLPs, (B) GII.10 P domain. Nano-42, Nano-14, Nano-26 were the strongest binders and detected GII.10 VLPs at a dilution of ~50 ng/ml. Nano-4 detected VLPs at a lower dilution of 0.1 μg/ml. Nano-32 detected VLPs at concentrations above 0.4 μg/ml and Nano-27 above 1.5 μg/ml. A similar binding pattern was observed with the GII.10 P domain, where Nano-42, Nano-4, Nano-14, Nano-26 detected P domain in concentrations up to 20 ng/ml. Nano-32, and Nano-27 reacted with the P domain at concentrations above 0.2 μg/ml. and 1.6 μg/ml respectively. (C-E) Nano-14, Nano-27 and Nano-32 bound only GII.10 P domain and showed no cross-reactivity to any other GII P domains (15 μg/ml) or to GI.1 and GI.11 VLPs (4 μg/ml). (TIF) [file ppat.1006636.s001.tif]

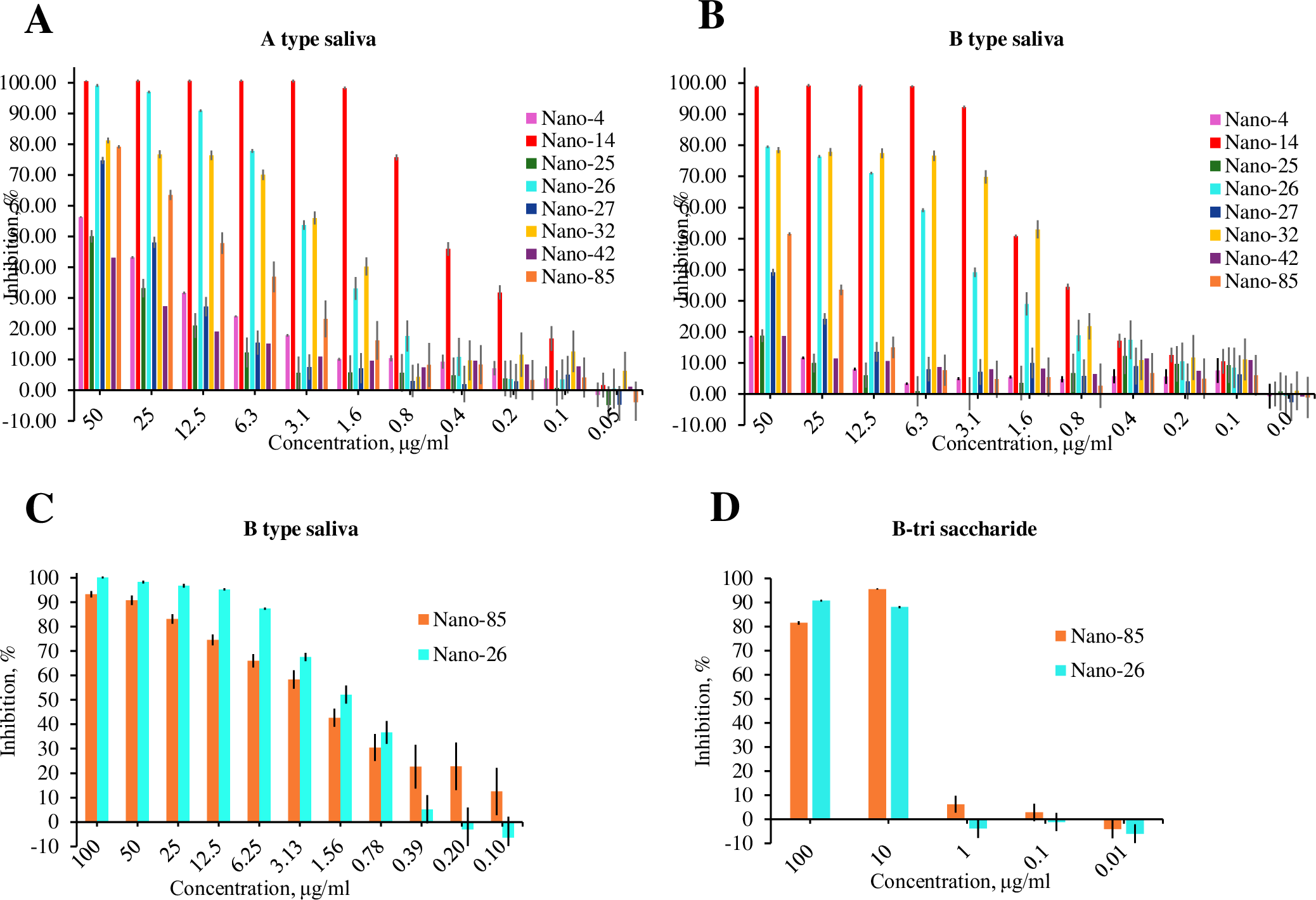

Supplement: S2 Fig — Saliva blocking assay with GII.10 VLPs (2.5 μg/ml) was performed similarly to PGM binding assay. (A) Nano-14, Nano-26, and Nano-32 inhibited 50% of the binding (IC50) to A type saliva at 0.4, 2.6, and 3.1 μg/ml, respectively. (B) For B type saliva IC50 values for Nano-14, Nano-26, and Nano-32 were 1.1, 4.3, and 1.8 μg/ml, respectively. Nano-85 showed only weak blocking potential. Binding was expressed as a percentage of the untreated VLP binding (100%). (C) Inhibition of GII.4 VLPs (0.5 μg/ml) binding to synthetic B-tri saccharide. Both Nano-26 and Nano-85 showed a complete inhibition at 10 μg/ml and no inhibition at 1 μg/ml. (D) Inhibition of GII.4 VLPs (0.5 μg/ml) binding to synthetic B type saliva. Nano-26 and Nano-85 blocked GII.4 VLP binding with IC50 of 0.7 and 1.2 μg/ml. All experiments were performed in triplicate (error bars are shown) and the cutoff was set at an OD490 of 0.15 (dashed line). (TIF) [file ppat.1006636.s002.tif]

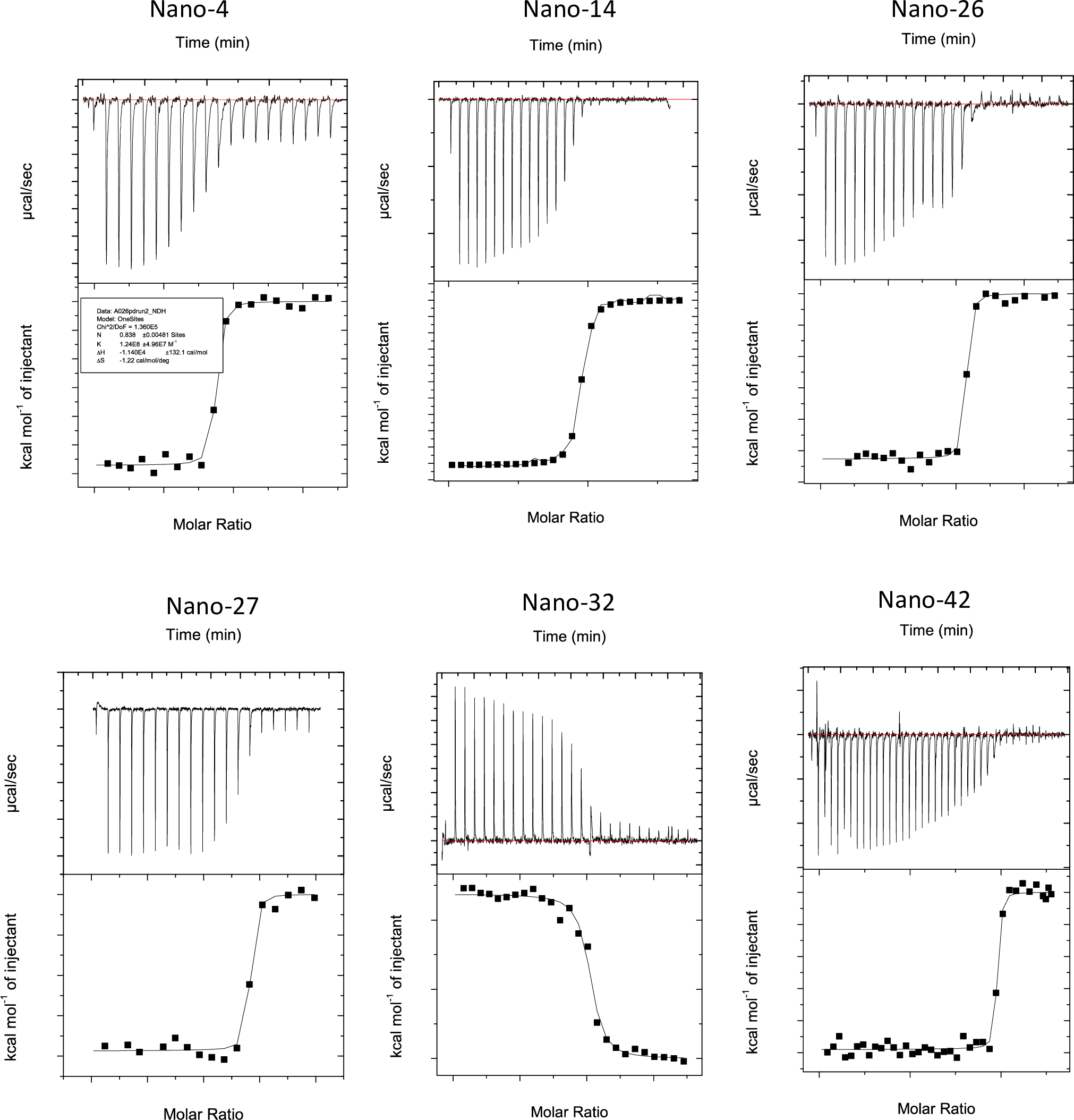

Supplement: S3 Fig — Titrations were performed at 25°C by injecting consecutive aliquots of 100–150 μM Nanobodies into 10–20 μM GII.10 P domain P domain. Examples of the titrations (upper panels) are shown. The binding isotherm was calculated using a single binding site model after subtraction of the heat of dilution (lower panels). Nano-32 binding to the P domain exhibited endothermic type of reaction, whereas all other Nanobodies showed exothermic binding reaction. (TIF) [file ppat.1006636.s003.tif]

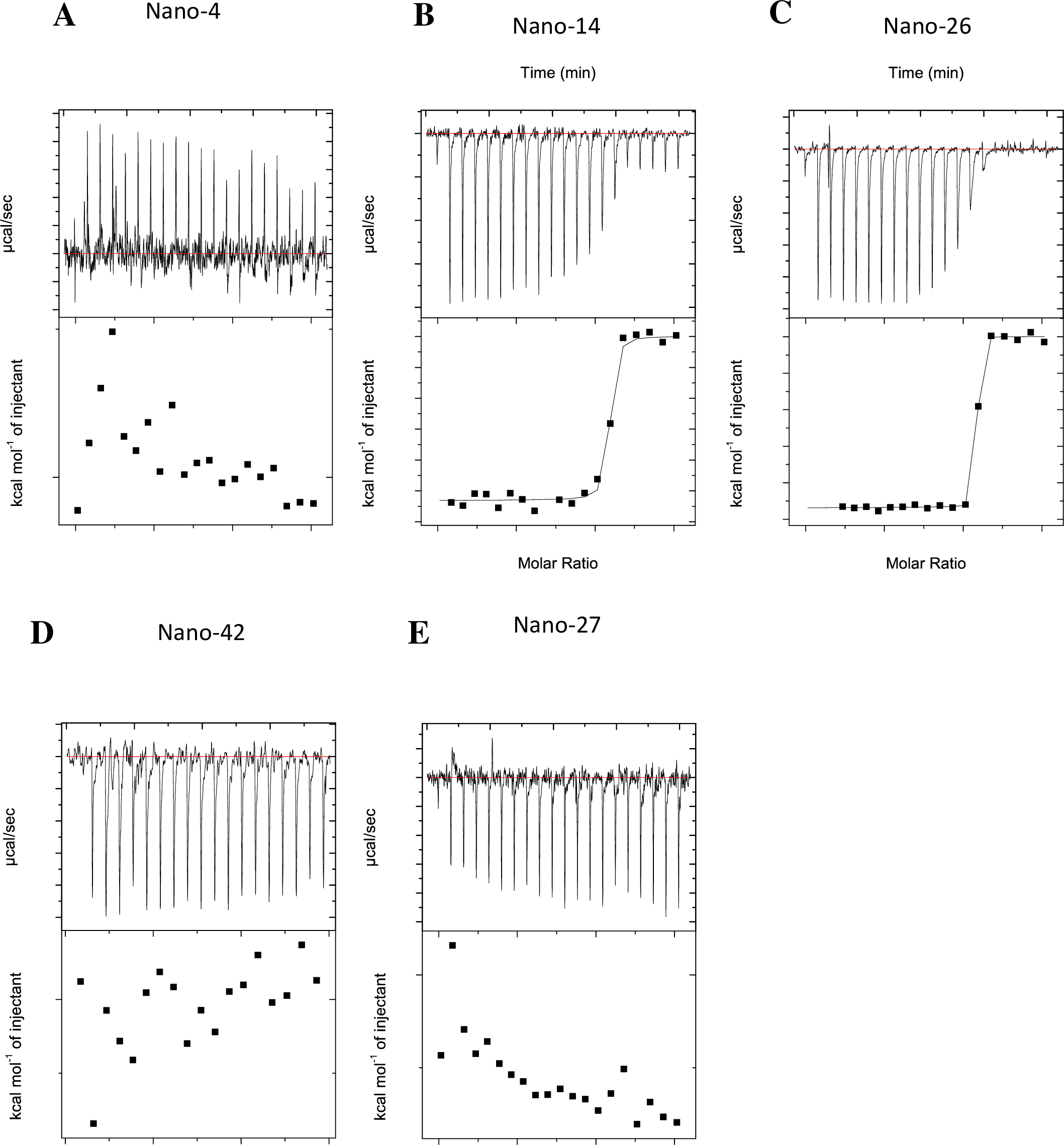

Supplement: S4 Fig — For the competitive ITC measurements, the P domain was pre-mixed with Nano-4, Nano-14, Nano-26, Nano-27, and Nano-42 in a 1:1 molar ratio. Standard titrations with Nano-85 were then performed. Titrations were done at 25°C by injecting consecutive aliquots of 100 μM Nanobody into 15 μM GII.10 P domain P domain. Examples of the titrations (upper panels) are shown. (A-C) Titration to P domain Nano-4, Nano-27 and Nano-42 showed the absence of heat release associated with injections, indicating the lack of binding. (D, E) Nano-85 showed the binding to GII.10 P domain Nano-26 and Nano-14 complexes with exothermic type of reaction, which resembled the binding of Nano-85 to P domain alone. The binding isotherm was calculated using a single binding site model after subtraction of the heat of dilution (lower panels). (TIF) [file ppat.1006636.s004.tif]

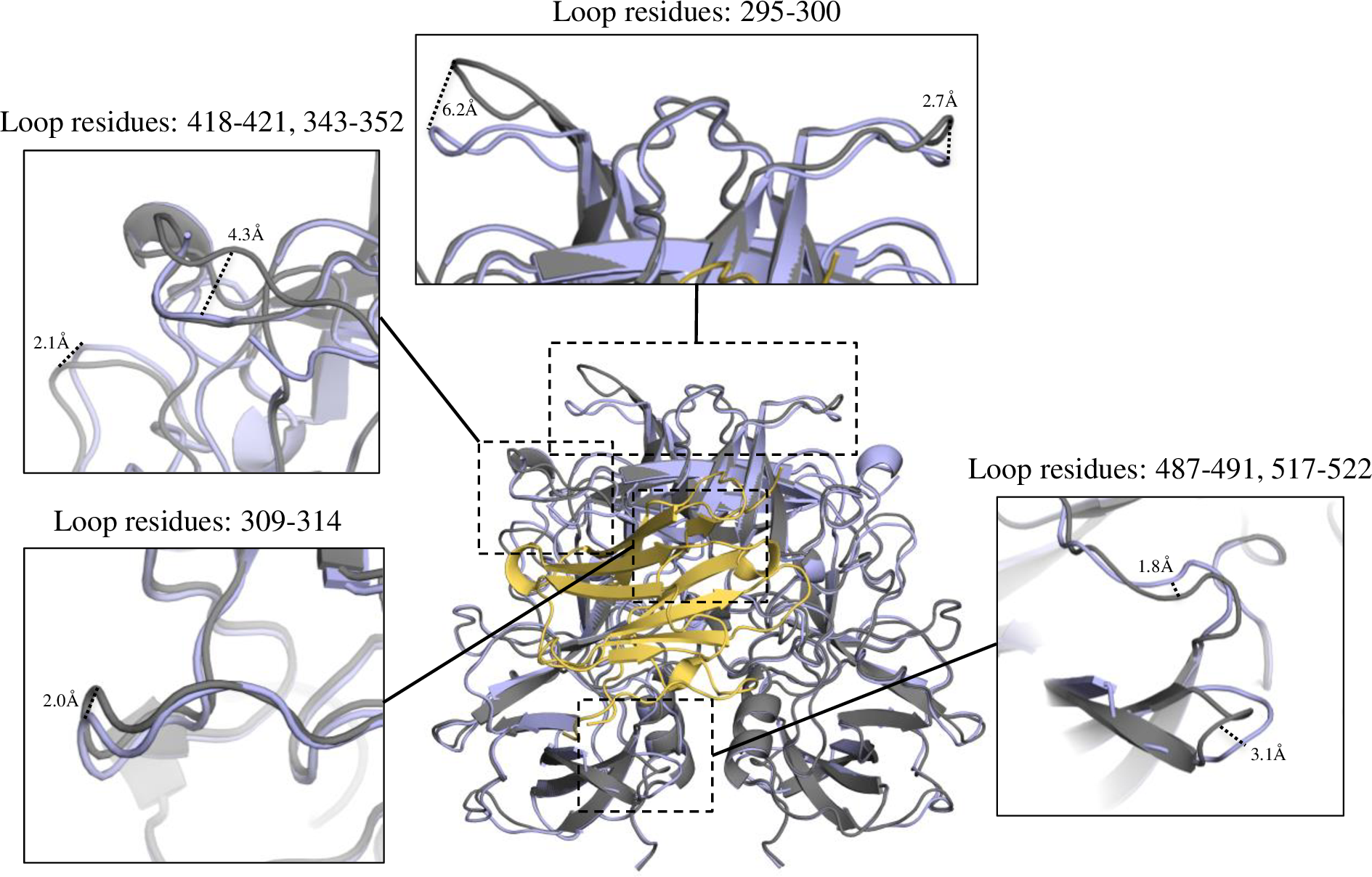

Supplement: S5 Fig — Several loops of GII.10 P domain in complex with Nano-32 had altered conformation compared to unliganded P domain. Loop between residues 295–300 was positioned symmetrically in both monomers which was not observed in apo-structure, but was characteristic for GII.10 P domain in complex with 30 mM B-tri saccharide (PDB code 4Z4Z). Loop 343–352 was partially disordered and deviated 4.3Å away from its position in unliganded structure. Loops 309–314, 418–420 as well as 487–491, 517–522 had slightly shifted conformation (2-3Å). (TIF) [file ppat.1006636.s005.tif]

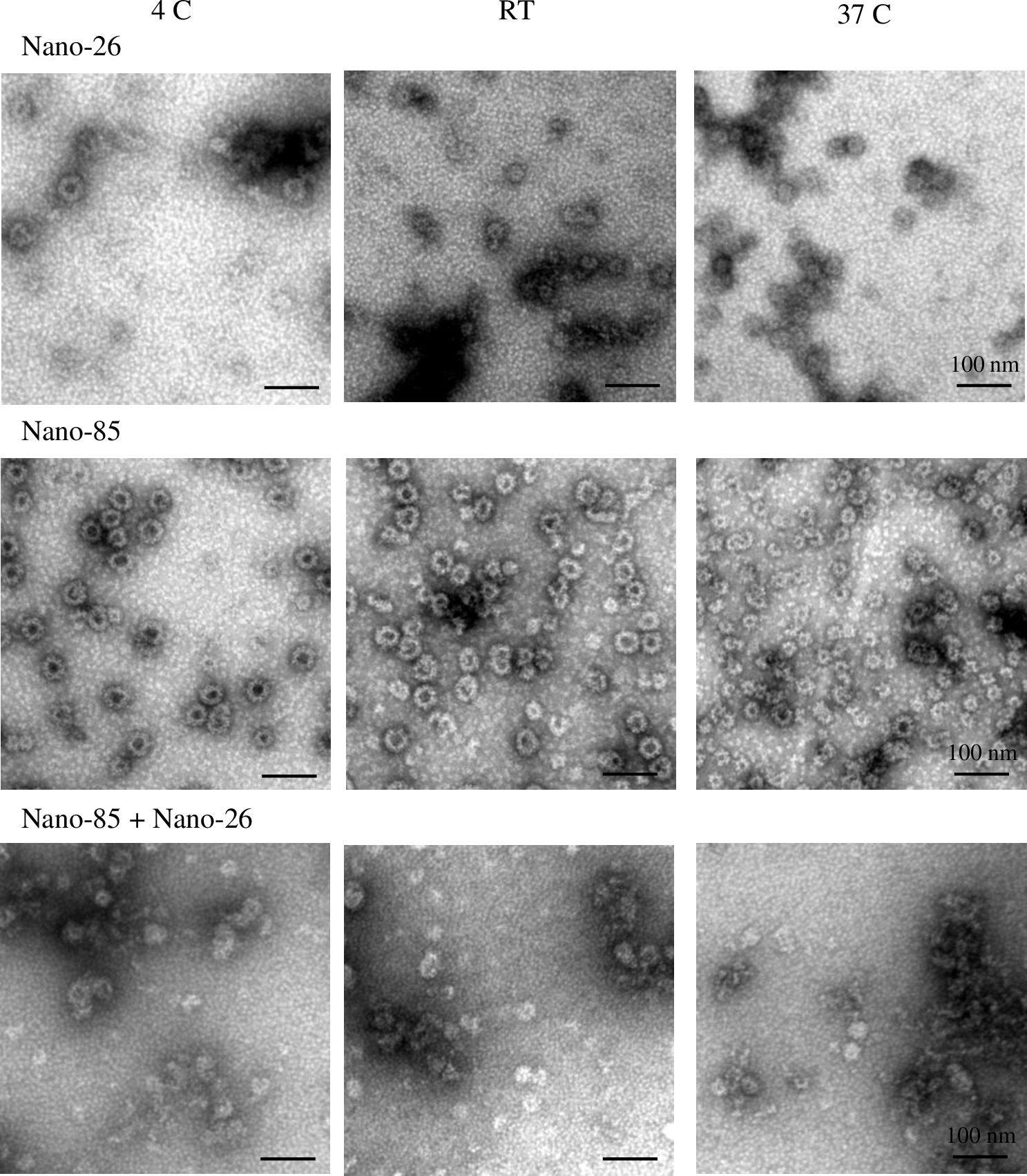

Supplement: S6 Fig — GII.10 VLPs were pre-incubated with Nano-85, Nano-26, or with both Nano-85 and Nano-26 for 30 min at 4°C, room temperature, and 37°C. After treatment VLPs were subjected to negative staining and examined by EM at 50,000 magnification. VLPs exposed to Nano-85 showed a temperature dependence of morphological changes. At 4°C a large portion of native 35–37 nm VLPs were visible, whereas at RT small 20–23 nm VLPs appeared and prevailed at 37°C. In case of Nano-26 and joint Nano-26 and Nano-85 treatment VLPs were largely degraded at any tested temperature. (TIF) [file ppat.1006636.s006.tif]

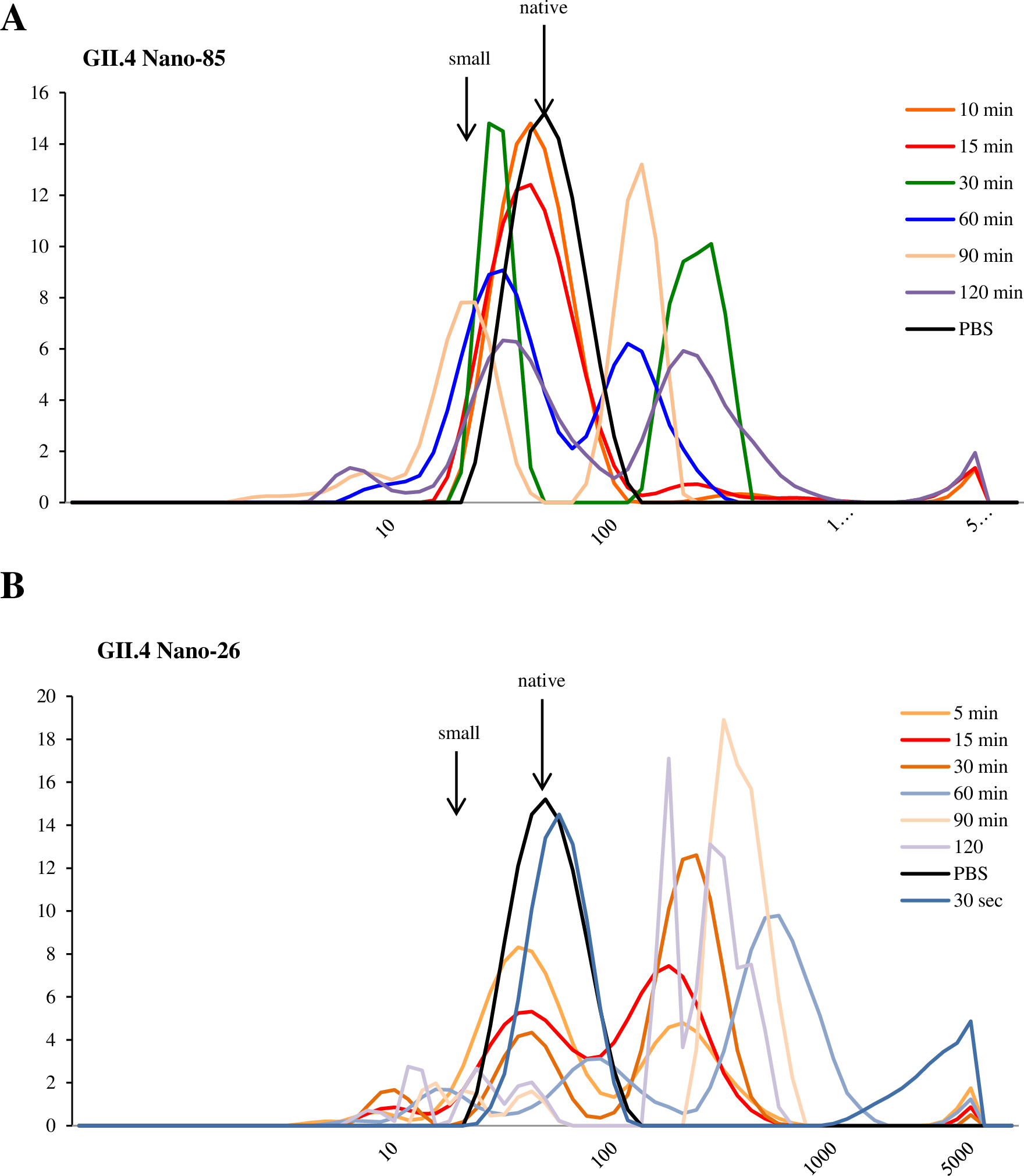

Supplement: S7 Fig — GII.4 VLPs were incubated with Nano-26 (A) or Nano-85 (B) for indicated periods of time at room temperature and DLS profiles were then measured. Peaks corresponding to large molecular weight aggregates appeared after 30 seconds in case of Nano-26 treated VLPs and 15 min for Nano-85. Arrows indicate native size VLPs (35–37 nm in diameter) and small VLPs (20–23 nm). (TIF) [file ppat.1006636.s007.tif]

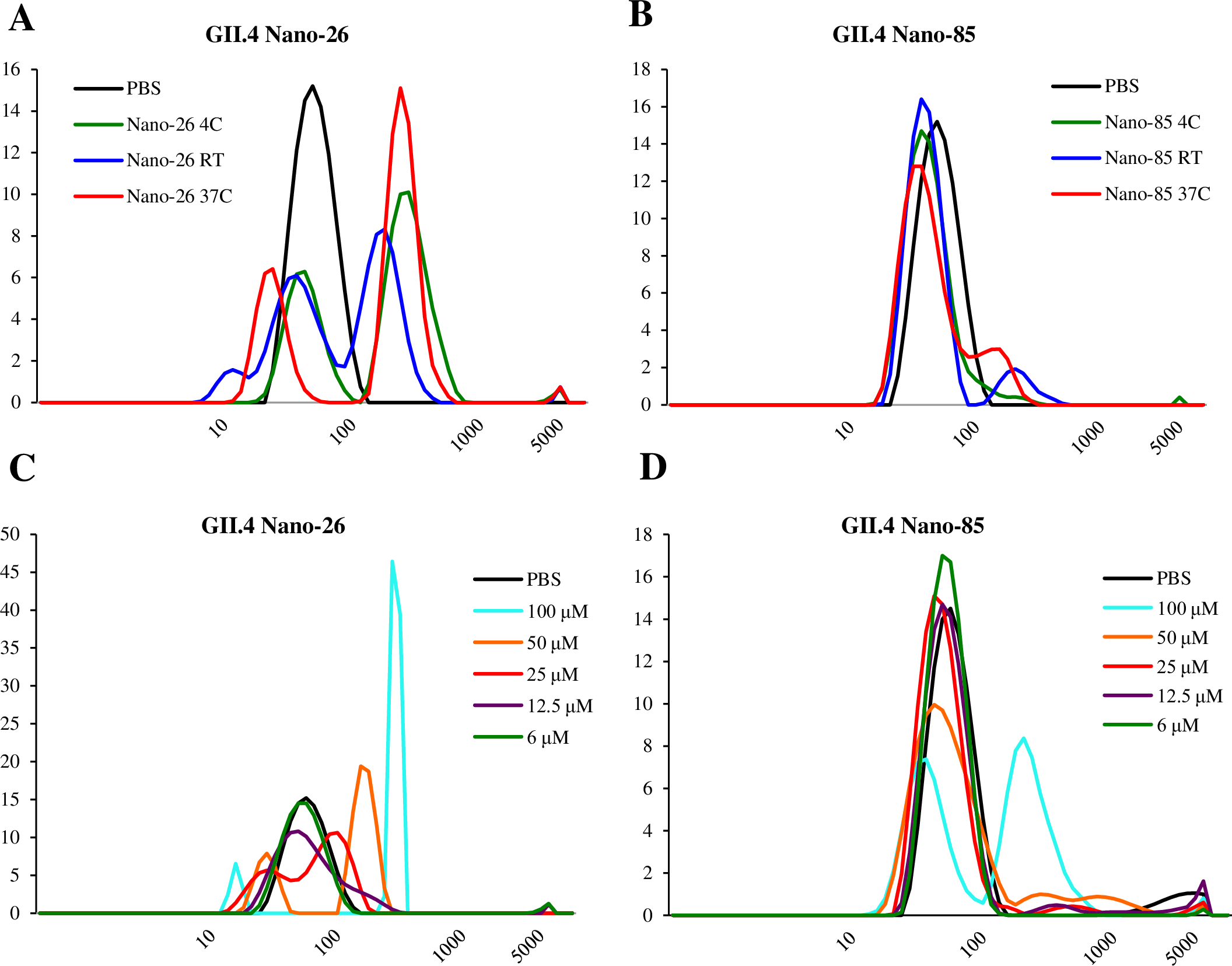

Supplement: S8 Fig — Hydrodynamic diameters of GII.4 VLPs treated with Nano-26 (A) or Nano-85 (B) at 4°C, RT, 37°C for 15 min were measured using DLS. VLPs had an increased particle heterogeneity at 37°C compared to 4°C and RT. Additionally, VLPs were pre-incubated with different concentrations of Nano-26 (C) or Nano-85 (D) for one hour at RT. Size distribution was altered at concentrations over 12.5 μM for Nano-26 and 50 μM for Nano-85. (TIF) [file ppat.1006636.s008.tif]

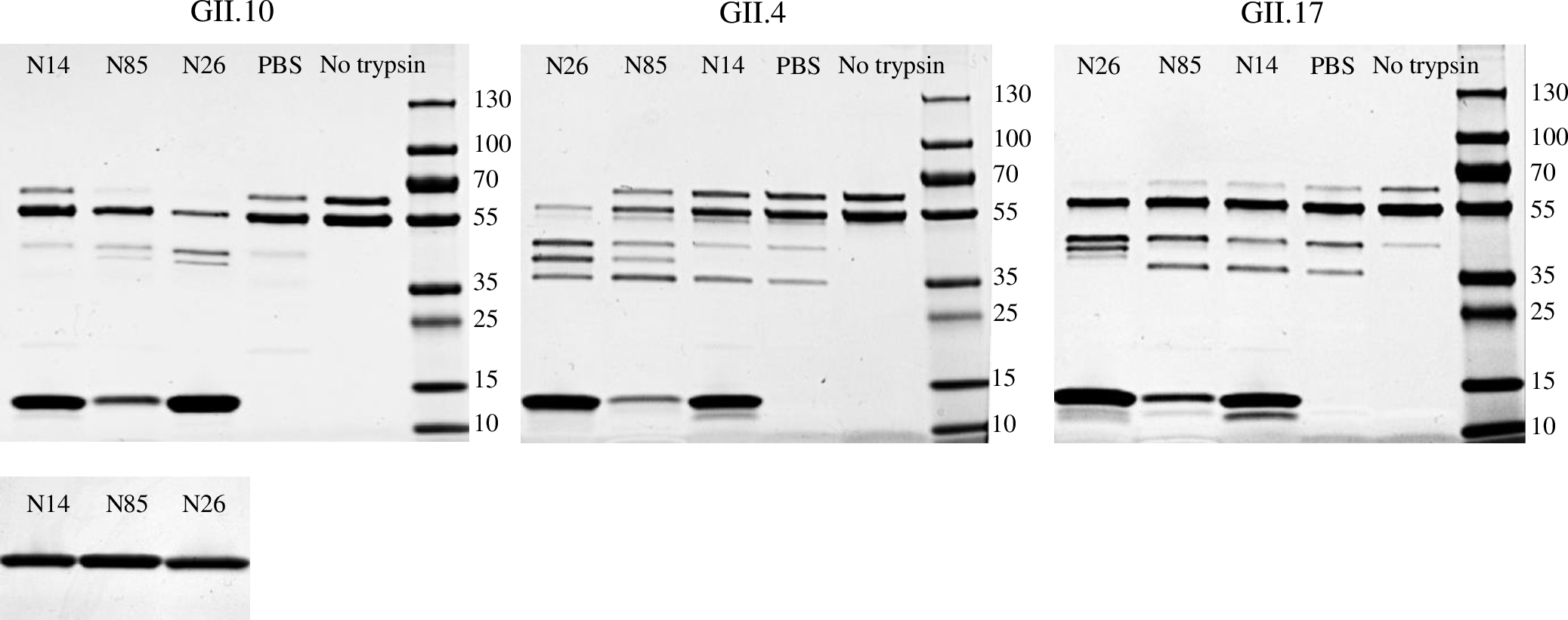

Supplement: S9 Fig — GII.10, GII.4, and GII.17 VLPs (1 mg/ml) were treated with 1 mg/ml of Nano-14 (lane N14), Nano-26 (lane N26), Nano-85 (lane N85), or PBS for 30 min at 37C. After the treatment samples were exposed to typsin (10 μg/ml final concentration) for additional 30 min at 37C. Samples were then run on the SDS PAGE and stained with Coomassie. VLPs without trypsin cleavage correspond to the last lane. Nano-14 does not impact the rate of protease degradation, whereas Nano-26 and Nano-85 significantly increased the digestion efficiency. (TIF) [file ppat.1006636.s009.tif]

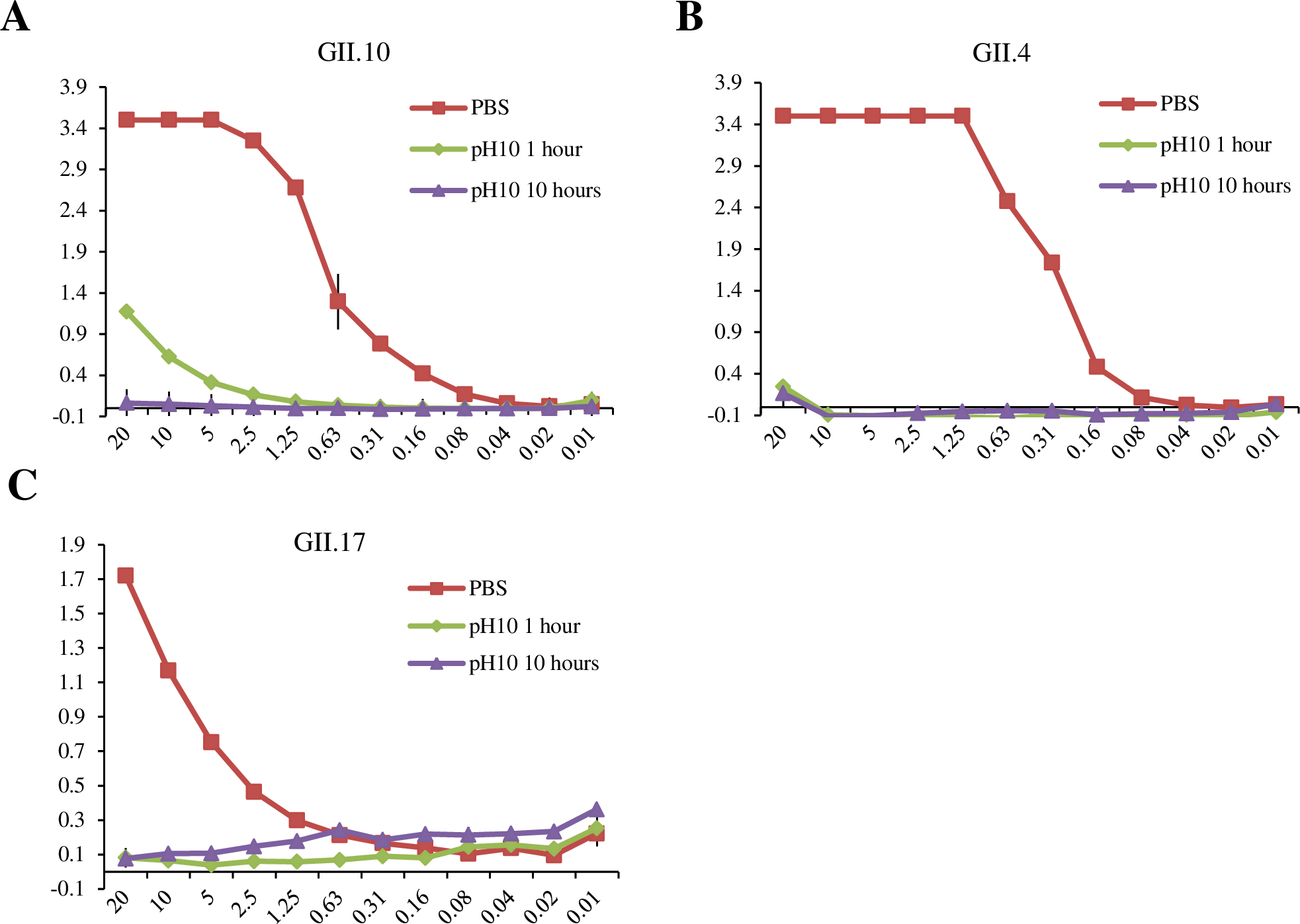

Supplement: S10 Fig — GII.10, GII.4, and GII.17 VLPs (A, B and C, respectively) were serially diluted in PBS, or 1M Tris buffer (pH 10), and incubated at room temperature for one or ten hours. Standard PGM binding assay was then performed using polyclonal serum against GII.10 for detection of GII.10 VLPs and polyclonal serum against GII.4 for detection of GII.4 and GII.17 VLPs. VLPs disassembled with pH 10 for ten hours showed no binding to PGM. Incubation with pH 10 for one hour greatly reduced the binding of GII.10 VLPs and completely abolished the binding of GII.4 and GII.17 VLPs. (TIF) [file ppat.1006636.s010.tif]
